# Gender-specific disaggregated analysis of childhood undernutrition in Ethiopia: evidence from 2000–2016 nationwide survey

**DOI:** 10.1186/s12889-023-16907-x

**Published:** 2023-10-19

**Authors:** Biniyam Sahiledengle, Lillian Mwanri, Cauane Blumenberg, Kingsley Emwinyore Agho

**Affiliations:** 1 Department of Public Health, Madda Walabu University Goba Referral Hospital, Bale-Goba, Ethiopia; 2 Research Centre for Public Health, Equity and Human Flourishing (PHEHF), Torrens University Australia, Adelaide Campus, SA 5000 Australia; 3 International Center for Equity in Health, Federal University of Pelotas, Pelotas, Brazil; 4 Post-Graduate Program in Epidemiology, Federal University of Pelotas, Pelotas, Brazil; 5 Causale Consultoria, Pelotas, Brazil; 6 School of Health Sciences, Western Sydney University, Locked Bag 1797, Penrith, NSW 2751 Australia; 7 School of Medicine, Translational Health Research Institute, Western Sydney University, Campbelltown Campus, Penrith, NSW 2571 Australia; 8 Faculty of Health Sciences, University of Johannesburg, Johannesburg, South Africa

**Keywords:** Sex differences, Gender inequality, Undernutrition, Stunting, Wasting, Underweight, Ethiopia

## Abstract

**Introduction:**

Childhood undernutrition has been investigated extensively in previous literature but gender inequality detailing the burden of undernutrition has not been adequately addressed in scientific papers, especially in Ethiopia, where undernutrition is known to be a public health problem of high significance, necessitating increased efforts to address it and reduce this inequality. This study was carried out to: (1) explore gender differences in the prevalence of stunting, wasting, and underweight, and (2) compare the factors associated with childhood undernutrition between boys and girls in Ethiopia.

**Methods:**

The study used a dataset of more than 33,564 children aged under 5 years (boys: 17,078 and girls: 16,486) who were included in the nationally representative Ethiopia Demographic and Health Survey (EDHS) from 2000 to 2016. The outcome variables were anthropometric indices: stunting (height-for-age < -2 standard deviations), wasting (weight-for-height < -2 standard deviations), and underweight (weight-for-age < -2 standard deviations). Gender-specific multilevel analyses were used to examine and compare the factors associated with child undernutrition.

**Results:**

The overall prevalence of stunting (49.1% for boys vs 45.3% for girls, *p* < 0.001), wasting (11.9% for boys vs 9.9% for girls, *p* < 0.001), and underweight (33.1% for boys vs 29.8% for girls, *p* < 0.001) higher among boys compared to girls. Boys significantly had higher odds of stunting (aOR: 1.31, 95%CI: 1.21–1.42), wasting (aOR: 1.35, 1.23–1.48), and underweight (aOR: 1.38, 95%CI: 1.26–1.50) than girls. The common factors associated with childhood undernutrition for male and female children were the child's age, perceived size of the child at birth, breastfeeding status, maternal stature, maternal education, toilet facility, wealth index, and place of residence. Boys who were perceived by their mothers to be average sized at birth and were born to uneducated mothers had a higher likelihood of experiencing wasting, in contrast to girls. Among boys, birth order (firstborn), household size (1–4), and place of residence (urban) were associated with lower odds of being underweight. Boys living in cities had lower odds of being stunted. While girls born to mothers with no education and worked in agriculture were at a higher odd of being stunted.

**Conclusion:**

Our study revealed that boys were more likely to be malnourished than girls, regardless of their age category, and there were variations in the factors determining undernutrition among boys and girls. The differences in the burden of undernutrition were significant and alarming, positioning Ethiopia to be questioned whether it will meet the set Sustainable Development Goals (SDGs), including SDG 2 of zero hunger by 2030. These findings call for more effort to address malnutrition as a significant public health issue in Ethiopia, and to urgently recognise the need for enhanced interventions that address the gender gap in childhood undernutrition.

**Supplementary Information:**

The online version contains supplementary material available at 10.1186/s12889-023-16907-x.

## Introduction

Undernutrition is associated with about 45% of all childhood deaths and continues to be of one of the greatest public health concerns in many low-middle-income (LMIC) countries [1]. Childhood undernutrition is known to be a cause of severe morbid and significant mortality rates in children and manifests in three broad forms including: wasting, stunting, and underweight. Stunting, which is the condition of being too short for a child’s age, undermines physical and cognitive development, increasing the risk of dying from common infections and predisposing them to non-communicable diseases later in life [2–5]. Wasting, which is defined as too low weight than expected for the child’s height, is an acute condition that can change frequently and rapidly over a calendar year [5]. Underweight is a composite index that accounts for both acute and chronic undernutrition [1, 2].

Globally in 2020, an estimated 149 million (22%) children under-five years of age were stunted, and 45 million (7%) were wasted [5]. The numbers show persistent regional disparities, with Africa bearing the heaviest burden of all forms of undernutrition. According to the World Health Organization (WHO), two out of five stunted children worldwide and over one-quarter of all wasted children under-five live in Africa [2]. Despite agreeing to work towards reversing all forms of malnutrition by 2030, the pace of necessary actions in Sub-Saharan African (SSA) countries, including Ethiopia, is still lower than acceptable [6, 7].

Ethiopia, a country long known for its significant burden of undernutrition, has been implementing a number of nutrition programs to combat undernutrition [7, 8], including the recent “*Seqota Declaration”* which aimed to end stunting in children under two years by 2030 [9]. Whilst impressive progress has been made in overall undernutrition in Ethiopia, with a decline in the prevalence of stunting from 51 to 37%, wasting from 12 to 7%, and underweight from 33 to 21% between 2005 and 2019 [10, 11], the reduction is not sufficient to meet the 2030 target. Additionally, studies have revealed the existence of gender disparities in undernutrition in Ethiopia and SSA countries, inequitably thwarting the expected progress [12–16].

A wide range of childhood undernutrition risk factors including maternal nutritional status and sociodemographic characteristics [17–21], environmental factors [19, 22, 23], and child related factors-such as the age of the child [17, 24, 25], size at birth [20, 21], complementary food starting before 6 months [26], lack of exclusive breastfeeding [18], and diarrhea [12, 18, 26–28] have been stated to be determinants of undernutrition. One of the children's risk factor is its sex [12, 25, 27, 29]. A systematic review and meta-analysis of sex differences in undernutrition that included 74 studies identified that boys had higher odds of being stunted, wasted, and underweight than girls [30]. Likewise, further analysis of the 2019 Ethiopia Mini Demographic and Health Survey (EMDHS) revealed significant gender disparities in the burden of childhood undernutrition in Ethiopia [31]. These findings, although focused on a limited set of indicators, showed that understanding the determinants of undernutrition in boys and girls is paramount.

There have been a number of studies on childhood undernutrition among Ethiopian children [32–35]. However, as far as is known, there is a scarcity of the evidence that identified the different risk factors of undernutrition comparing boys and girls in Ethiopia. The only study that could be found was the one by Samuel and colleagues who focused on gender differences in nutritional status [36], and which was also limited to infants aged between 6 and 11 months. This study also investigated stunting and wasting, and was localized in two regions, hence limiting its generalisability at the national level [36]. Another study by Wang et al. [37] used a decomposition technique to evaluate the differences in nutritional outcomes by observable factors and socio-economic characteristics using the Ethiopia Demographic and Health Survey (EDHS) 2011. Apart from these two studies, past studies in Ethiopia have mainly focused on urban–rural inequalities [31], socioeconomic inequality [32–34, 37], and many concentrated on the spatial analysis of one or more forms of undernutrition [19, 23, 38–40, 40, 41]. For example, although Negasi (2021) used the Ethiopia Socioeconomic Survey (ESS) to investigate inequalities in childhood undernutrition, their focus was on socioeconomic status [33], Jember et al. (2021) [42], and Tesema et al. [38] focused on geospatial inequality of anaemia among children. These studies provided insight about forms of inequality, however, non- as focused on gender inequalities. Thus, it is important to gain an understanding of the undernutrition inequalities that exist between boys and girls, as well as to identify potential risk factors that lead to such differences during their first five years of life.

To fill this gap of the literature, our aim was twofold as follows, to: (i) describe the prevalence of childhood undernutrition, specifically targeting it common but important forms, i.e., stunting, wasting and underweight among boys and girls, and (ii) identify their risk factors according to the gender of the child. This study will contribute and improve the dearth of information and existing data gaps in gender inequality in undernutrition in Ethiopia. Additionally, the study also will contribute evidence that will enhance information towards the global commitment of "*Leaving no one behind*" from the Sustainable Development Goals (SDG) agenda Goal 2 target 2.2.

## Methods

### Data sources and sampling design

This analysis used data from four rounds of the Ethiopia Demographic and Health Survey (EDHS) conducted in 2000, 2005, 2011, and 2016 [10, 43–45]. Ethiopia Demographic and Health Survey is a nationally representative cross-sectional household survey, being also representative at regional and area of residence (urban/rural) levels. The EDHS sampling and household listing methods are described elsewhere [45]. We included children aged 0–59 months born to mothers aged 15–49 years. Children's height and weight measurements were obtained, and anthropometric indices (height-for-age, weight-for-height, and weight-for-age) were calculated. Children were excluded if they lacked anthropometric measures or had implausible values (values < -6 SDs and >  + 6 SDs). We used information from 33,564, 33,583, and 33,729 children (weighted sample) who were born 5 years before the surveys were included to analyze stunting, wasting, and underweight, respectively.

### Outcome variables

The outcome variables of the study were stunting, wasting, and being underweight. Height-for-age (HAZ), weight-for-height (WHZ), and weight-for-age (WAZ) z-scores below − 2 SDs of the WHO Child Growth Standard were used to define stunting, wasting, and underweight, respectively [46].

### Independent variables

The independent variables were selected based on previous literature [47–53]. The selected variables were classified into three categories according to the UNICEF’s conceptual framework of malnutrition: immediate (individual-level factors), underlying (household-level factors), and basic (community-level factors) determinants [54]. The individual-level determinants consisted of child characteristics (age in months, birth order, birth interval, size at birth, breastfeeding status, diarrhea and fever in the past 2 weeks) and maternal characteristics (age, education, occupation, stature, listening to the radio, and watching television). The household-level covariates included wealth index, household number of residents, type of toilet facility, source of drinking water, and time to get to a water source. The community-level factors include the place of residence (urban or rural), contextual region of residence (agrarian, pastoralist, and city administration), and survey year. Variable descriptions for various independent variables are described in Supplementary File 1.

The household wealth index was calculated using the principal components analysis method. Households are given scores based on the number and kinds of consumer goods they own, ranging from a television to a bicycle or car, in addition to housing characteristics such as source of drinking water, toilet facilities, and flooring materials. The wealth index was categorized into wealth quintiles: 'very poor', 'poor', 'middle', 'rich' and 'very rich [45]. For this analysis, we re-coded the wealth index into three categories for adequate sampling in each category: 'poor' (poor and very poor), 'middle' and 'rich' (rich and very rich) [22].

### Data analysis

All analyses were carried out using STATA/MP version 14.1 (StataCorp, College Station, TX, USA). The survey command (*svy*) was used to take into account the sampling design of the survey. Descriptive statistics such as absolute and relative frequencies were used to present the distribution of all variables and stratified by sex (male or female). A comparison between undernutrition indicators according to child’s sex was carried out. The Chi-squared test and a 95% confidence interval (CI) were used to compare the prevalence of stunting, wasting, and underweight by sex. Then, differences in the prevalence of stunting, wasting, and underweight by gender were presented using *equiplot* graphs, which visually represent absolute inequalities. These graphs make it easy for non-specialists to grasp the idea of gender inequalities (https://equidade.org/equiplot).

Gender-specific disaggregated analyses were conducted to identify factors associated with outcome variables. Given the hierarchical nature of the EDHS data, a two-level multilevel binary logistic regression model was built with individuals (level 1) nested within communities (level 2). Accordingly, four models were constructed in this analysis. The empty model (*Model I*) was fitted without explanatory variables to estimate random variation in the intercept. *Model II* was constructed to examine the effects of individual-level characteristics, *Model III* was fitted to assess the effects of community-level characteristics, and *Model IV* adjusted for the individual- and community-level variables simultaneously. Separate models were run for boys and girls for stunting, wasting, and underweight to explore the individual and community-level factors associated with child undernutrition according to sex. We present the disaggregated analysis results for girls and boys in a condensed table. In the model-building process, we first performed unadjusted bivariate multilevel models disaggregated by the sex for each pair of outcome and covariate. Variables in bivariable analysis with a *p*-value < 0.2 and known confounder variables were entered into the multilevel multivariate logistic regression model. A multicollinearity test was performed among independent variables, and no evidence of multicollinearity was found. The intraclass correlation coefficient (ICC) was computed for each model to evaluate whether the variation in outcome variable is primarily within or between communities. The adjusted odds ratio (AOR) with a 95% Confidence Interval (CI) was estimated. A significance level of 5% was considered in all analyses.

## Results

### Characteristics of the sample

Table 1 presents the weighted proportion of demographic characteristics of the study sample. In all datasets, almost 49.0% of under-five children were girls. Most of the children were born to mothers with no schooling (72.8%), rural residents (89.3%), and poor households (45.4%). Occurrence of diarrhea was slightly higher among boys (17.4% vs. 16.3%, *p* = 0.002), and more girls were small at birth (34.1% vs. 25.3%, *p* < 0.001). Breastfeeding initiation within one hour of post-partum was similar between boys and girls (53.7% vs. 54.8%, *p* = 0.108).

**Table 1:** Characteristics associated with undernutrition among children 0–59 months by sex, (EDHS 2000–2016, *n* = 33,564)^a,b^

Variables	Number of under-five children, (n)	Weighted %	Child’s sex		
			**Boys (%)**	**Girls (%)**	***p*-value**^**c**^
**Overall prevalence of undernutrition**					
Stunting	15,878	47.3	49.18	45.36	*p* < 0.001
Wasting	3,679	10.9	11.95	9.92	*p* < 0.001
Underweight	10,627	31.5	33.13	29.83	*p* < 0.001
***Child factors***					
**Age (months)**					0.859
< 6	3,394	10.1	10.6	10.3	
6–11	3,540	10.5	9.6	10.4	
12–23	6,604	19.6	18.6	19.3	
24–35	6,429	19.1	19.2	18.6	
36–59	13,693	40.7	41.9	41.4	
**Birth order**					0.062
Firstborn	6,012	17.9	18.7	18.4	
2–4	14,551	43.1	42.2	43.1	
5 or higher	13,165	39.0	39.1	38.5	
**Birth interval**					0.265
< 33 months	23,274	69.0	68.5	69.8	
≥ 33 months	10,455	31.0	31.5	30.2	
**Size at birth**					*p* < 0.001
Larger	10,420	31.0	35.9	27.1	
Average	13,228	38.3	38.8	38.8	
Small	9,982	29.7	25.3	34.1	
**Currently breastfeeding**					0.009
Yes	25,045	74.2	70.1	71.8	
No	8,684	25.8	29.9	28.2	
**Early initiation of breastfeeding (*n* = 25,988)**					0.108
Yes	14,172	54.5	53.7	54.8	
No	11,816	45.5	46.3	45.2	
**Full vaccination (*n* = 28,852)**					0.676
Yes	5,595	19.4	18.6	18.9	
No	23,257	80.6	81.4	81.1	
**Diarrhea**					0.002
Yes	5,769	17.1	17.4	16.3	
No	27,912	82.9	82.6	83.7	
**Fever**					0.070
Yes	6,857	20.4	20.7	19.6	
No	26,822	79.6	79.3	80.4	
***Parental factors***					
**Mother’s age**					0.196
< 18	279	0.8	0.9	0.8	
18–24	7,820	23.2	24.0	23.4	
25–34	17,123	50.8	49.4	51.1	
≥ 35	8,506	25.2	25.7	24.7	
**Mother’s education**					0.897
No education	24,567	72.8	73.8	73.3	
Primary and above	9,162	27.2	26.2	26.7	
**Mother’s occupation**					0.007
Not working	16,411	48.8	49.5	48.5	
Non agriculture	7,058	21.0	20.6	21.4	
Agriculture	10,156	30.2	29.9	30.1	
**Maternal stature**					0.729
Normal/Tall (> = 155 cm)	20,575	61.0	60.1	61.0	
Short (145 to 154.9 cm)	12,366	36.7	37.5	36.4	
Very short (< 145 cm)	787	2.3	2.4	2.5	
**Listening to radio**					0.417
Yes	11,567	34.3	34.0	34.4	
Not at all	22,152	65.7	66.0	65.6	
**Watching television**					0.857
Yes	5,893	17.5	17.3	16.8	
Not at all	27,815	82.5	82.7	83.1	
***Household factors***					
**Wealth index**					0.367
Poor	10,862	45.4	45.6	45.7	
Middle	4,991	20.8	21.3	20.6	
Rich	8,085	33.8	33.1	33.7	
**Household size**					0.091
1–4	8,114	24.1	26.2	25.7	
≥ 5	25,615	75.9	73.8	74.3	
**Toilet facility**					0.631
Improved	3,769	11.4	11.2	11.4	
Unimproved	29,370	88.6	88.8	88.6	
**Source of drinking water**					0.845
Improved	12,797	38.6	38.7	38.5	
Unimproved	20,335	61.4	61.3	61.5	
**Time to get a water source**					0.186
On-premise	1,766	5.3	5.3	5.2	
≤ 30 min	19,532	58.3	57.3	58.8	
31–60 min	6,900	20.6	21.1	19.8	
> 60 min	5,324	15.9	16.3	16.2	
***Community-level characteristics***					
**Residence**					0.816
Urban	3,624	10.7	10.5	10.9	
Rural	30,105	89.3	89.5	89.1	
**Region**					0.058
Agrarian	18,366	54.5	54.2	55.2	
Pastoralist	14,574	43.2	43.5	42.5	
City administration	789	2.3	2.3	2.3	

^a^The absolute number of under-five children is weighted

^b^EDHS-2000 = 9,789; EDHS-2005 = 4,287; EDHS-2011 = 9,991; EDHS-2016 = 9,496

^c^Pearson’s chi-squared test was used to compute the p-values for men v. female contrast

### Prevalence of stunting

Table 1 also shows the weighted prevalence of child undernutrition and gender-based inequalities. The overall prevalence of stunting among children under the age of five years was 47.3% (95%CI: 46.8–47.8). The overall prevalence of stunting was higher among male than female children (49.2% vs. 45.4%, *p* < 0.001).

### Prevalence of wasting

**Fig. 1:**
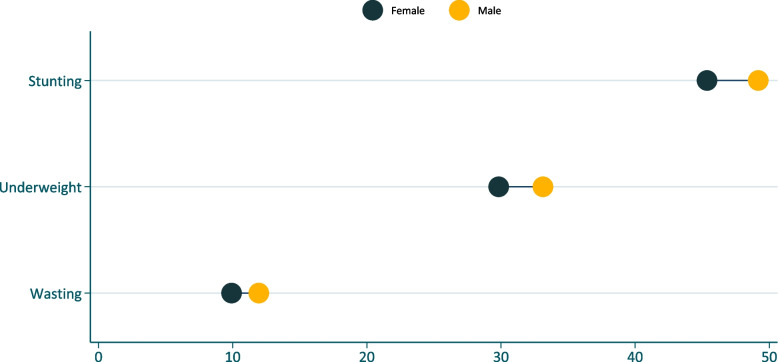
Inequality in child undernutrition by sex in Ethiopia

The overall prevalence of wasting among children under the age of five years was 10.9% (95%CI: 10.6–11.3). The prevalence of wasting was also higher among boys than girls (11.95% vs. 9.92%, *p* < 0.001) (Fig. 1).

### Prevalence of underweight

The overall prevalence of underweight among children under the age of five years was 31.5% (31.0–32.0). The prevalence of underweight was also higher among boys than girls (33.13% vs. 29.83%, *p* < 0.001) (Fig. 1).

### Prevalence of stunting, wasting and underweight by gender

**Fig. 2:**
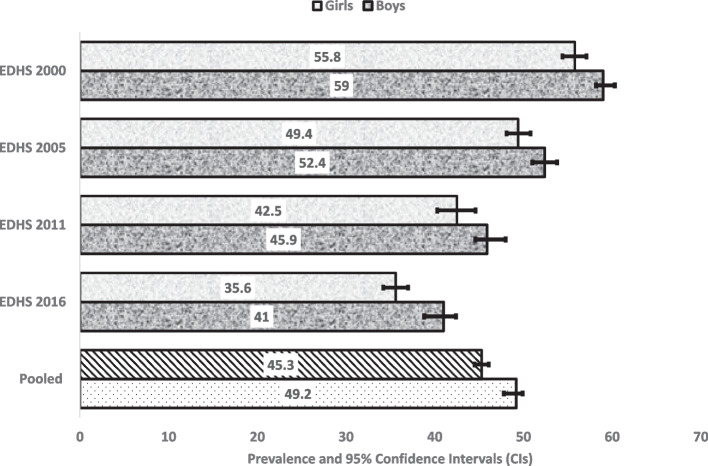
Stunning in different EDHS survey years, by sex in Ethiopia

**Fig. 3:**
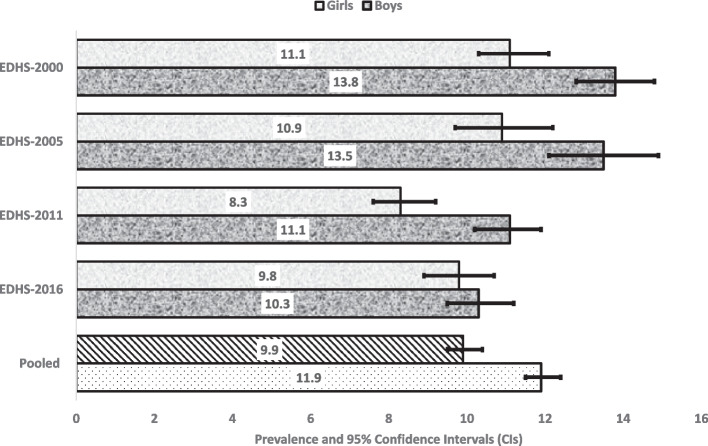
Wasting in different EDHS survey years, by sex in Ethiopia

**Fig. 4:**
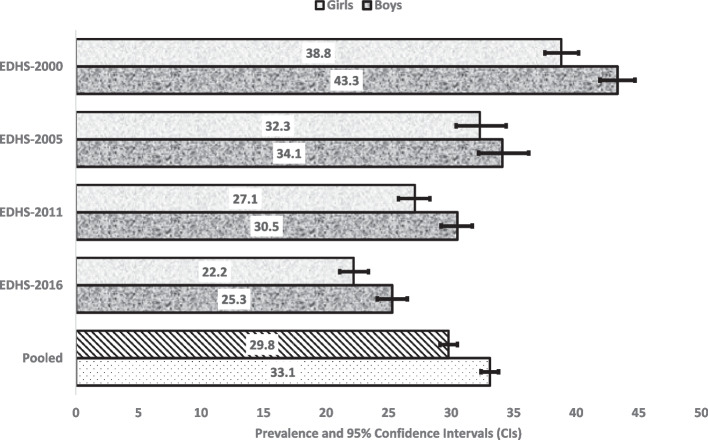
Underweight in different EDHS survey years, by sex in Ethiopia

**Fig. 5:**
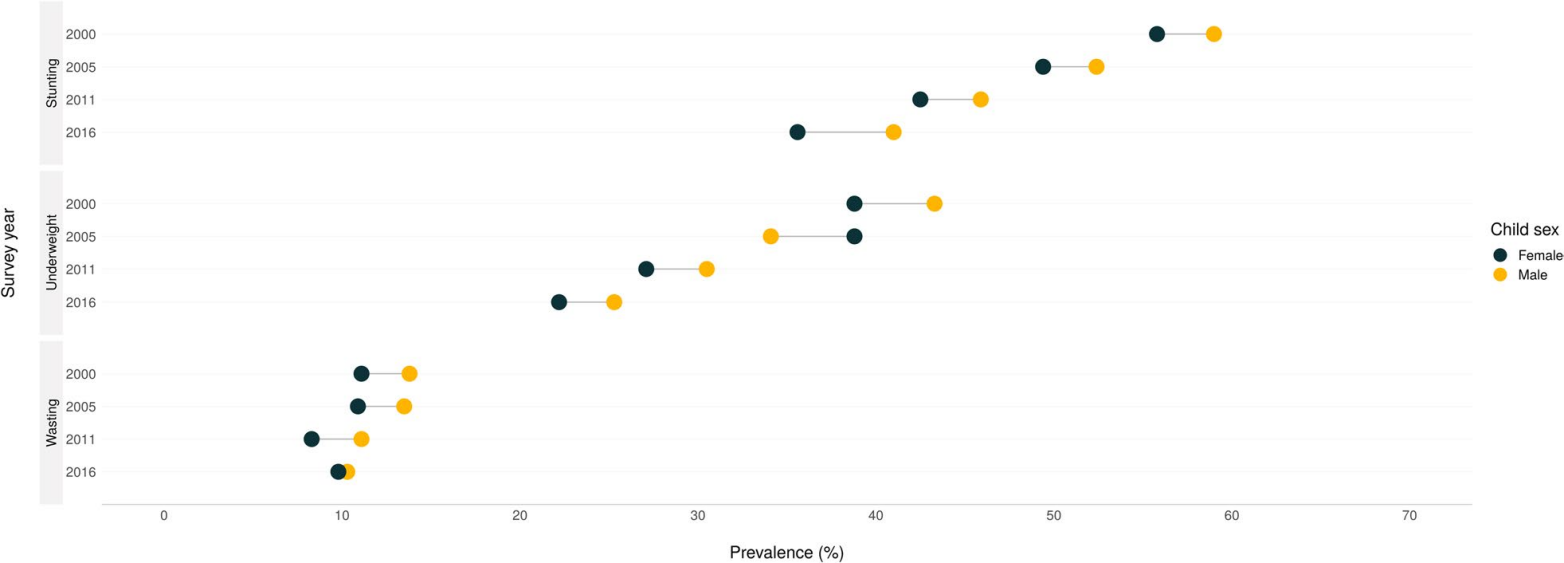
Equiplots showing child undernutrition in different EDHS survey years, by sex in Ethiopia

Figures 2, 3 and 4 reported 16 years prevalence of stunting, wasting and underweight and their 95% CIs by gender. Overall, the prevalence stunting, wasting and underweight seem to has been reduced in the past 16 years with girls reporting lower prevalence than boys (Fig. 5). Compared to EDHS-2000, the prevalence of stunting and underweight has also been reduced significantly, this was not the same for the prevalence of wasting which appeared to have been reduced but did not differ statistically to that of EDHS-2016 (Fig. 3 and Supplementary File 2).

**Fig. 6:**
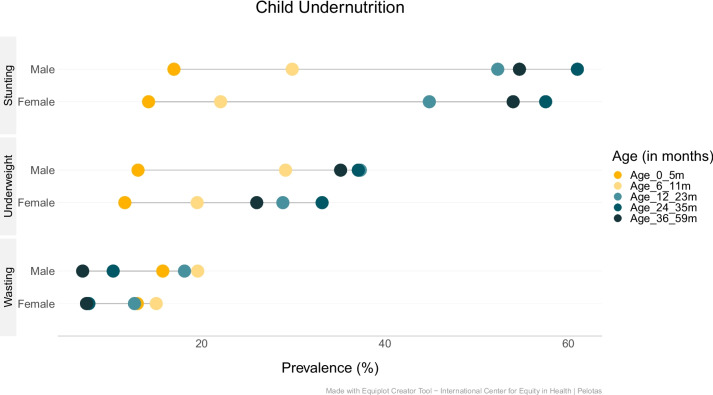
Equiplots showing child undernutrition among boys and girls, by age in Ethiopia

The differences in the prevalence of undernutrition between boys and girls stratified by EDHS survey year and age are presented in Fig. 6. Across all age groups (i.e., < 6 months, 6–11, 12–23, 24–35, and 36–59 months) male children had a higher prevalence of stunting, wasting and underweight compared to girls (Supplementary Files 3, 4 and 5).

### Factors associated with stunting, wasting and underweight in male and female children

The results of the multilevel bivariable logistic regression analysis for stunting, wasting, and underweight for boys and girls are shown in Tables 2, 3 and 4.

**Table 2:** Factors associated with stunting among boys and girls in children 0–59 months, EDHS 2000–2016

Variables	Boys (*n* = 17,078)			Girls (*n* = 16,486)		
	**Stunting proportion (%)**	**Unadjusted OR (95%CI)**	***p*-value**	**Stunting proportion (%)**	**Unadjusted OR (95%CI)**	***p*-value**
***Child factors***						
**Age (months)**						
< 6	16.98	0.15 (0.13–0.17)	*p* < 0.001	14.21	0.13 (0.11–0.15)	*p* < 0.001
6–11	29.88	0.29 (0.26–0.33)	*p* < 0.001	22.07	0.22 (0.19–0.25)	*p* < 0.001
12–23	52.30	0.93 (0.85–1.02)	0.147	44.84	0.71 (0.65–0.78)	*p* < 0.001
24–35	60.99	1.26 (1.15–1.37)	*p* < 0.001	57.53	1.23 (1.12–1.34)	*p* < 0.001
36–59	54.67	Ref.		53.98	Ref.	
**Birth order**						
Firstborn	47.44	0.76 (0.69–0.83)	*p* < 0.001	41.62	0.72 (0.65–0.79)	*p* < 0.001
2–4	48.52	0.84 (0.78–0.91)	*p* < 0.001	44.77	0.86 (0.79–0.92)	*p* < 0.001
5 or higher	50.69	Ref.		47.73	Ref.	
**Birth interval**						
< 33 months	48.44	Ref.		44.21	Ref.	
≥ 33 months	50.79	1.14 (1.06–1.22)	*p* < 0.001	48.00	1.18 (1.09–1.27)	*p* < 0.001
**Size of a child at birth**						
Larger	44.87	Ref.		41.37	Ref.	
Average	48.27	1.14 (1.06–1.23)	*p* < 0.001	43.39	1.09 (1.01–1.19)	0.039
Small	56.78	1.54 (1.41–1.67)	*p* < 0.001	50.76	1.45 (1.33–1.58)	*p* < 0.001
**Currently breastfeeding**						
Yes	50.72	Ref.		47.40	Ref.	
No	48.63	1.13 (1.05–1.21)	*p* < 0.001	44.67	1.18 (1.09–1.27)	*p* < 0.001
**Early initiation of breastfeeding**						
Yes	47.22	Ref.		42.67	Ref.	
No	50.94	1.15 (1.07–1.24)	*p* < 0.001	48.05	1.14 (1.05–1.23)	0.001
**Full vaccination**						
Yes	52.08	Ref.		47.33	Ref.	
No	48.67	0.93 (0.86–1.01)	0.101	45.08	0.93 (0.86–1.02)	0.123
**Diarrhea**						
Yes	53.52	1.30 (1.19–1.42)	*p* < 0.001	49.84	1.19 (1.09–1.30)	*p* < 0.001
No	48.27	Ref.		44.51	Ref.	
**Fever**						
Yes	51.78	1.19 (1.10–1.29)	*p* < 0.001	47.48	1.19 (1.10–1.29)	*p* < 0.001
No	48.52	Ref.		44.82	Ref.	
***Parental factors***						
**Mother's age**						
< 18	45.75	0.67 (0.45–0.99)	0.049	38.37	0.49 (0.34–0.73)	*p* < 0.001
18–24	47.73	0.81 (0.74–0.89)	*p* < 0.001	42.17	0.77 (0.69–0.84)	*p* < 0.001
25–34	49.10	0.88 (0.82–0.96)	0.003	45.59	0.89 (0.82–0.97)	0.006
≥ 35	50.77	Ref.		48.05	Ref.	
**Mother's education**						
No education	52.55	1.80 (1.67–1.94)	*p* < 0.001	48.15	1.78 (1.65–1.92)	*p* < 0.001
Primary and above	40.13	Ref.		38.87	Ref.	
**Mother's occupation**						
Not working	47.32	Ref.		41.96	Ref.	
Non agriculture	46.19	0.89 (0.82–0.97)	0.011	42.53	1.01 (0.92–1.09)	0.920
Agriculture	54.27	1.49 (1.38–1.62)	*p* < 0.001	52.61	1.64 (1.51–1.77)	*p* < 0.001
**Maternal stature**						
Normal/Tall	43.25	Ref.		40.44	Ref.	
Short	57.96	1.79 (1.66–1.92)	*p* < 0.001	52.35	1.69 (1.57–1.81)	*p* < 0.001
Very short	65.63	2.61 (2.06–3.31)	*p* < 0.001	64.69	2.86 (2.25–3.63)	*p* < 0.001
**Listening to radio**						
Yes	55.74	Ref.		41.20	Ref.	
Not at all	51.73	1.32 (1.23–1.42)	*p* < 0.001	47.53	1.33 (1.24–1.43)	*p* < 0.001
**Watching television**						
Yes	39.89	Ref.		36.34	Ref.	
Not at all	51.17	1.97 (1.81–2.15)	*p* < 0.001	47.24	1.90 (1.74–2.08)	*p* < 0.001
***Household factors***						
**Wealth index**						
Poor	48.89	Ref.		46.01	Ref.	
Middle	45.59	0.91 (0.81–1.01)	0.080	41.55	0.88 (0.78–0.98)	0.028
Rich	39.79	0.59 (0.54–0.64)	*p* < 0.001	34.09	0.58 (0.53–0.64)	*p* < 0.001
**Household size**						
1–4	47.59	0.88 (0.82–0.95)	0.002	42.35	0.84 (0.79–0.93)	*p* < 0.001
≥ 5	49.68	Ref.		46.30	Ref.	
**Toilet facility**						
Improved	39.59	Ref.		37.06	Ref.	
Unimproved	50.34	1.98 (1.80–2.17)	*p* < 0.001	46.48	1.90 (1.73–2.09)	*p* < 0.001
**Source of drinking water**						
Improved	44.49	Ref.		42.58	Ref.	
Unimproved	52.02	1.98 (1.80–2.17)	*p* < 0.001	47.17	1.31 (1.22–1.40)	*p* < 0.001
**Time to get a water source**						
On-premise	30.45	Ref.		22.46	Ref.	
≤ 30 min	51.44	2.77 (2.42–3.17)	*p* < 0.001	46.14	2.81 (2.43–3.24)	*p* < 0.001
31–60 min	48.38	2.75 (2.37–3.18)	*p* < 0.001	48.58	2.90 (2.48–3.39)	*p* < 0.001
> 60 min	48.76	2.65 (2.28–3.07)	*p* < 0.001	45.83	2.88 (2.46–3.36)	*p* < 0.001
***Community-level characteristics***						
**Residence**						
Urban	36.55	0.43 (0.39–0.47)	*p* < 0.001	33.08	0.45 (0.41–0.49)	*p* < 0.001
Rural	50.67	Ref.		46.87	Ref.	
**Region**						
Agrarian	52.65	Ref.		49.16	Ref.	
Pastoralist	46.13	0.94 (0.87–1.02)	0.145	41.53	0.96 (0.88–1.04)	0.302
City administration	26.11	0.49 (0.44–0.54)	*p* < 0.001	25.49	0.57 (0.52–0.64)	*p* < 0.001

**Table 3:** Factors associated with wasting among boys and girls in children 0–59 months, EDHS 2000–2016

Variables	Boys (*n* = 17,112)			Girls (*n* = 16,471)		
	**Wasting proportion (%)**	**Unadjusted OR (95%CI)**	***p*-value**	**Wasting proportion (%)**	**Unadjusted OR (95%CI)**	***p*-value**
***Child factors***						
**Age (months)**						
< 6	15.76	2.21 (1.89–2.59)	*p* < 0.001	12.98	2.01 (1.69–2.37)	*p* < 0.001
6–11	19.57	2.73 (2.35–3.17)	*p* < 0.001	15.05	2.32 (1.98–2.73)	*p* < 0.001
12–23	18.13	2.29 (2.02–2.61)	*p* < 0.001	12.67	1.89 (1.66–2.18)	*p* < 0.001
24–35	10.35	1.32 (1.14–1.51)	*p* < 0.001	7.70	1.01 (0.86–1.19)	0.849
36–59	7.02	Ref.		7.44	Ref.	
**Birth order**						
Firstborn	10.99	0.72 (0.63–0.83)	*p* < 0.001	7.88	0.74 (0.64–0.86)	*p* < 0.001
2–4	11.45	0.87 (0.78–0.96)	*p* < 0.001	9.92	0.85 (0.76–0.95)	0.004
5 or higher	12.90	Ref.		10.84	Ref.	
**Birth interval**						
< 33 months	11.77	Ref.		9.74	Ref.	
≥ 33 months	12.31	0.99 (0.89–1.09)	0.896	10.33	1.04 (0.92–1.15)	0.516
**Size of a child at birth**						
Larger	9.46	Ref.		7.71	Ref.	
Average	11.59	1.27 (1.14–1.43)	*p* < 0.001	8.92	1.12 (0.97–1.28)	0.119
Small	16.03	1.87 (1.66–2.11)	*p* < 0.001	12.81	1.81 (1.58–2.07)	*p* < 0.001
**Currently breastfeeding**						
Yes	13.32	Ref.		10.72	Ref.	
No	8.09	0.68 (0.61–0.76)	*p* < 0.001	7.55	0.69 (0.62–0.78)	*p* < 0.001
**Early initiation of breastfeeding**						
Yes	12.70	1.05 (0.94–1.16)	0.370	10.08	Ref.	
No	14.08	Ref.		10.87	1.02 (0.91–1.15)	0.682
**Full vaccination**						
Yes	10.76	Ref.		6.88	Ref.	
No	13.25	1.48 (1.30–1.69)	*p* < 0.001	10.91	1.82 (1.57–2.12)	*p* < 0.001
**Diarrhea**						
Yes	17.11	1.61 (1.44–1.81)	*p* < 0.001	14.54	1.67 (1.48–1.89)	*p* < 0.001
No	10.82	Ref.		8.98	Ref.	
**Fever**						
Yes	16.95	1.49 (1.34–1.65)	*p* < 0.001	13.19	1.63 (1.45–1.83)	*p* < 0.001
No	10.63	Ref.		9.10	Ref.	
***Parental factors***						
**Mother's age**						
< 18	15.22	1.55 (0.95–2.51)	0.075	8.99	1.24 (0.75–2.05)	0.400
18–24	12.72	1.05 (0.92–1.19)	0.459	9.85	0.98 (0.85–1.114)	0.857
25–34	11.35	0.95 (0.85–1.07)	0.430	9.58	0.89 (0.79–1.01)	0.076
≥ 35	12.28	Ref.		10.72	Ref.	
**Mother's education**						
No education	12.82	1.58 (1.41–1.76)	*p* < 0.001	10.97	1.58 (1.39–1.79)	*p* < 0.001
Primary and above	9.57	Ref.		7.09	Ref.	
**Mother's occupation**						
Not working	11.80	Ref.		9.98	Ref.	
Non agriculture	10.67	0.73 (0.64–0.82)	*p* < 0.001	7.90	0.75 (0.65–0.86)	*p* < 0.001
Agriculture	13.11	0.95 (0.85–1.06)	0.356	11.24	1.10 (0.98–1.24)	0.100
**Maternal stature**						
Normal/Tall	11.36	Ref.		9.72	Ref.	
Short	12.73	1.08 (0.98–1.19)	0.387	10.13	0.96 (0.86–1.07)	0.536
Very short	14.65	1.15 (0.84–1.58)	0.126	11.83	0.83 (0.57–1.23)	0.371
**Listening to radio**						
Yes	9.78	Ref.		8.17	Ref.	
Not at all	13.05	1.47 (1.32–1.63)	*p* < 0.001	10.83	1.59 (1.42–1.79)	*p* < 0.001
**Watching television**						
Yes	8.80	Ref.		6.19	Ref.	
Not at all	12.62	1.86 (1.62–2.14)	*p* < 0.001	10.71	1.98 (1.69–2.32)	*p* < 0.001
***Household factors***						
**Wealth index**						
Poor	14.23	Ref.		11.11	Ref.	
Middle	10.27	0.68 (0.58–0.81)	*p* < 0.001	10.28	0.86 (0.72–1.02)	0.094
Rich	7.66	0.49 (0.43–0.57)	*p* < 0.001	6.61	0.53 (0.45–0.61)	*p* < 0.001
**Household size**						
1–4	12.52	0.91 (0.82–1.02)	0.102	8.79	0.84 (0.74–0.95)	0.005
≥ 5	11.76	Ref.		10.27	Ref.	
**Toilet facility**						
Improved	9.60	Ref.		6.68	Ref.	
Unimproved	12.21	1.49 (1.30–1.73)	*p* < 0.001	10.35	1.67 (1.42–1.96)	*p* < 0.001
**Source of drinking water**						
Improved	11.22	Ref.		9.84	Ref.	
Unimproved	12.36	1.25 (1.13–1.37)	*p* < 0.001	9.99	1.13 (1.01–1.25)	0.026
**Time to get a water source**						
On-premise	8.81	Ref.		6.50	Ref.	
≤ 30 min	11.92	1.51 (1.23–1.85)	*p* < 0.001	9.92	1.73 (1.36–2.18)	*p* < 0.001
31–60 min	12.42	1.73 (1.39–2.15)	*p* < 0.001	9.97	1.82 (1.41–2.34)	*p* < 0.001
> 60 min	12.45	1.94 (1.56–2.41)	*p* < 0.001	11.29	2.21 (1.72–2.84)	*p* < 0.001
***Community-level characteristics***						
**Residence**						
Urban	8.58	0.65 (0.56–0.75)	*p* < 0.001	5.97	0.59 (0.50–0.70)	*p* < 0.001
Rural	12.34	Ref.		10.41	Ref.	
**Region**						
Agrarian	12.53	Ref.		10.42	Ref.	
Pastoralist	11.50	0.98 (0.88–1.10)	0.811	9.53	0.95 (0.88–1.04)	0.302
City administration	6.58	0.63 (0.54–0.74)	*p* < 0.001	5.37	0.57 (0.51–0.64)	*p* < 0.001

**Table 4:** Factors associated with underweight among boys and girls in children 0–59 months, EDHS 2000–2016

Variables	Boys (*n* = 17,188)			Girls (*n* = 16,541)		
	**Underweight proportion (%)**	**Unadjusted OR (95%CI)**	***p*-value**	**Underweight proportion (%)**	**Unadjusted OR (95%CI)**	***p*-value**
***Child factors***						
**Age (months)**						
< 6	13.06	0.29 (0.25–0.34)	*p* < 0.001	11.62	0.23 (0.19–0.27)	*p* < 0.001
6–11	29.15	0.68 (0.61–0.78)	*p* < 0.001	19.51	0.45 (0.40–0.52)	*p* < 0.001
12–23	37.31	1.08 (0.99–1.19)	0.073	28.86	0.79 (0.71–0.86)	*p* < 0.001
24–35	37.09	1.17 (1.07–1.28)	0.001	33.14	0.94 (0.86–1.04)	0.251
36–59	35.16	Ref.		26.01	Ref.	
**Birth order**						
Firstborn	29.28	0.64 (0.58–0.71)	*p* < 0.001	25.08	0.64 (0.58–0.72)	*p* < 0.001
2–4	32.82	0.84 (0.78–0.91)	*p* < 0.001	29.62	0.82 (0.76–0.89)	*p* < 0.001
5 or higher	35.20	Ref.		32.23	Ref.	
**Birth interval**						
< 33 months	32.17	Ref.		28.42	Ref.	
≥ 33 months	35.20	1.13 (1.05–1.21)	0.001	33.03	1.21 (1.12–1.31)	*p* < 0.001
**Size of a child at birth**						
Larger	26.64	Ref.		25.41	Ref.	
Average	31.86	1.28 (1.18–1.39)	*p* < 0.001	29.21	1.15 (1.05–1.26)	0.003
Small	44.36	2.12 (1.94–2.32)	*p* < 0.001	39.82	1.85 (1.69–2.04)	*p* < 0.001
**Currently breastfeeding**						
Yes	33.58	Ref.		29.87	Ref.	
No	31.85	0.96 (0.89–1.04)	0.388	29.69	1.01 (0.93–1.09)	0.824
**Early initiation of breastfeeding**						
Yes	31.28	Ref.		28.20	Ref.	
No	36.17	1.14 (1.05–1.23)	0.001	31.92	1.15 (1.06–1.25)	0.001
**Full vaccination**						
Yes	33.28	Ref.		27.86	Ref.	
No	33.86	1.16 (1.06–1.27)	0.001	30.37	1.24 (1.13–1.36)	*p* < 0.001
**Diarrhea**						
Yes	17.11	1.53 (1.41–1.67)	*p* < 0.001	14.54	1.52 (1.38–1.66)	*p* < 0.001
No	10.82	Ref.		8.98	Ref.	
**Fever**						
Yes	40.70	1.44 (1.32–1.56)	*p* < 0.001	35.86	1.45 (1.34–1.58)	*p* < 0.001
No	31.18	Ref.		28.30	Ref.	
***Parental factors***						
**Mother's age**						
< 18	32.35	0.75 (0.49–1.13)	0.178	29.46	0.64 (0.42–0.97)	0.036
18–24	30.43	0.80 (0.73–0.88)	*p* < 0.001	25.53	0.73 (0.66–0.81)	*p* < 0.001
25–34	33.58	0.94 (0.87–1.03)	0.198	30.28	0.87 (0.79–0.95)	0.002
≥ 35	34.73	Ref.		32.82	Ref.	
**Mother's education**						
No education	36.48	2.11 (1.94–2.29)	*p* < 0.001	33.15	2.13 (1.95–2.32)	*p* < 0.001
Primary and above	24.09	Ref.		20.91	Ref.	
**Mother's occupation**						
Not working	32.43	Ref.		28.20	Ref.	
Non agriculture	30.26	0.75 (0.69–0.83)	*p* < 0.001	25.48	0.87 (0.79–0.95)	0.005
Agriculture	36.38	1.29 (1.19–1.39)	*p* < 0.001	35.40	1.48 (1.36–1.61)	*p* < 0.001
**Maternal stature**						
Normal/Tall	28.60	Ref.		26.41	Ref.	
Short	39.80	1.60 (1.49–1.72)	*p* < 0.001	34.42	1.47 (1.37–1.59)	*p* < 0.001
Very short	45.68	2.21 (1.75–2.78)	*p* < 0.001	46.76	2.37 (1.88–2.99)	*p* < 0.001
**Listening to radio**						
Yes	28.15	Ref.		25.49	Ref.	
Not at all	35.71	1.55 (1.44–1.67)	*p* < 0.001	32.08	1.54 (1.43–1.67)	*p* < 0.001
**Watching television**						
Yes	23.10	Ref.		21.44	Ref.	
Not at all	35.26	2.34 (2.11–2.58)	*p* < 0.001	31.56	2.29 (2.06–2.55)	*p* < 0.001
***Household factors***						
**Wealth index**						
Poor	34.38	Ref.		31.23	Ref.	
Middle	28.69	0.76 (0.67–0.85)	*p* < 0.001	26.03	0.81 (0.72–0.92)	0.001
Rich	21.90	0.42 (0.38–0.47)	*p* < 0.001	19.30	0.45 (0.40–0.49)	*p* < 0.001
**Household size**						
1–4	30.83	0.78 (0.72–0.84)	*p* < 0.001	27.38	0.84 (0.77–0.91)	*p* < 0.001
≥ 5	33.85	Ref.		30.59	Ref.	
**Toilet facility**						
Improved	24.66	Ref.		22.32	Ref.	
Unimproved	34.15	2.23 (2.01–2.48)	*p* < 0.001	30.73	2.12 (1.89–2.38)	*p* < 0.001
**Source of drinking water**						
Improved	29.81	Ref.		26.74	Ref.	
Unimproved	35.15	1.49 (1.39–1.61)	*p* < 0.001	31.67	1.39 (1.29–1.51)	*p* < 0.001
**Time to get a water source**						
On-premise	17.68	Ref.		13.27	Ref.	
≤ 30 min	34.17	2.70 (2.30–3.16)	*p* < 0.001	30.00	2.98 (2.50–3.55)	*p* < 0.001
31–60 min	33.67	2.80 (2.36–3.32)	*p* < 0.001	31.56	3.14 (2.60–3.78)	*p* < 0.001
> 60 min	34.08	2.99 (2.53–3.55)	*p* < 0.001	32.92	3.76 (3.12–4.52)	*p* < 0.001
***Community-level characteristics***						
**Residence**						
Urban	21.72	Ref.		17.04	Ref.	
Rural	34.47	0.36 (0.32–0.40)	*p* < 0.001	31.39	0.36 (0.32–0.40)	*p* < 0.001
**Region**						
Agrarian	35.85	Ref.		33.18	Ref.	
Pastoralist	30.87	0.98 (0.91–1.07)	0.769	26.49	0.95 (0.86–1.04)	0.269
City administration	12.30	0.45 (0.40–0.51)	*p* < 0.001	11.45	0.49 (0.44–0.55)	*p* < 0.001

Table 5 and Supplementary File 6 present the condensed tables for multilevel multivariable analysis results. Overall, boys significantly had higher odds of stunting (AOR: 1.31, 95%CI: 1.21–1.42), wasting (AOR: 1.35, 1.23–1.48), and underweight (AOR: 1.38, 95%CI: 1.26–1.50) than girls.

**Table 5:** Multivariable multilevel models on factors associated with stunting, wasting, and underweight among boys and girls in children 0–59 months, EDHS 2000–2016 (Condensed model)

Variables	Stunting		Wasting		Underweight	
	**Boys (*n* = 17,078)**	**Girls (*n* = 16,486)**	**Boys (*n* = 17,112)**	**Girls (*n* = 16,471)**	**Boys (*n* = 17,188)**	**Girls (*n* = 16,541)**
	**AOR (95%CI%)**	**AOR (95%CI%)**	**AOR (95%CI%)**	**AOR (95%CI%)**	**AOR (95%CI%)**	**AOR (95%CI%)**
***Child factors***						
**Age (months)**						
< 6	0.06 (0.05–0.08)^**^	0.08 (0.06–0.11)^**^	2.12 (1.68–2.68)^**^	1.77 (1.37–2.29)^**^	0.21 (0.16–0.26)^**^	0.17 (0.13–0.22)^**^
6–11	0.14 (0.11–0.18)^**^	0.12 (0.09–0.16)^**^	2.37 (1.88–2.97)^**^	1.84 (1.44–2.36)^**^	0.49 (0.39–0.62)^**^	0.30 (0.23–0.37)^**^
12–23	0.54 (0.43–0.66)^**^	0.47 (0.38–0.58)^**^	2.08 (1.71–2.54)^**^	1.66 (1.34–2.06)^**^	0.88 (0.73–1.06)	0.61 (0.50–0.73)^**^
24–35	1.13 (0.93–1.36)	1.14 (0.95–1.38)	1.29 (1.06–1.58)^*^	0.93 (0.74–1.17)	1.18 (0.98–1.43)	0.91 (0.75–1.09)
36–59	Ref.	Ref.	Ref.	Ref.	Ref.	Ref.
**Birth order**						
Firstborn	0.98 (0.77–1.25)	1.20 (0.94–1.54)	0.78 (0.60–1.01)	0.85 (0.63–1.13)	0.77 (0.59–0.98)^*^	0.82 (0.63–1.07)
2–4	0.88 (0.75–1.03)	1.04 (0.88–1.22)	0.89 (0.75–1.06)	0.95 (0.79–1.14)	0.85 (0.72–1.01)	0.93 (0.78–1.11)
5 or higher	Ref.	Ref.	Ref.	Ref.	Ref.	Ref.
**Birth interval**						
< 33 months	Ref.	Ref	-	-	Ref	Ref
≥ 33 months	0.87 (0.66–1.15)	0.89 (0.68–1.18)			0.87 (0.66–1.16)	1.03 (0.77–1.36)
**Size of a child at birth**						
Larger	Ref.	Ref.	Ref.	Ref.	Ref.	Ref.
Average	1.31 (1.15–1.50)^**^	1.17 (1.01–1.36)^*^	1.27 (1.09–1.49)^*^	1.03 (0.85–1.25)	1.36 (1.18–1.57)^**^	1.22 (1.04–1.44)^*^
Small	1.80 (1.55–2.09)^**^	1.57 (1.35–1.83)^**^	1.73 (1.46–2.03)^**^	1.55 (1.29–1.87)^**^	2.46 (2.11–2.89)^**^	1.94 (1.65–2.29)^**^
**Currently breastfeeding**						
Yes	Ref.	Ref.	Ref.	Ref.	-	-
No	0.66 (0.56–0.78)^**^	0.71 (0.60–0.83)^**^	1.03 (0.87–1.21)	0.99 (0.82–1.19)		
**Early initiation of breastfeeding**						
Yes	Ref.	Ref.	-	-	Ref	Ref
No	1.02 (0.90–1.14)	1.01 (0.89–1.14)			0.93 (0.82–1.05)	1.01 (0.88–1.14)
**Full vaccination**						
Yes	Ref.	Ref.	Ref.	Ref.	Ref.	Ref.
No	1.04 (0.90–1.19)	1.01 (0.88–1.17)	1.09 (0.92–1.29)	1.24 (1.02–1.52)^*^	1.06 (0.92–1.23)	1.21 (1.03–1.41)^*^
**Diarrhea**						
Yes	1.16 (0.99–1.35)	1.04 (0.88–1.22)	1.25 (1.06–1.48)^*^	1.25 (1.03–1.51)^*^	1.26 (1.08–1.47)^*^	1.32 (1.12–1.56)^*^
No	Ref.	Ref.	Ref.	Ref.	Ref.	Ref.
**Fever**						
Yes	0.99 (0.86–1.15)	1.02 (0.87–1.19)	1.20 (1.02–1.41)^*^	1.48 (1.25–1.77)^**^	1.14 (0.99–1.33)	1.18 (1.01–1.38)^*^
No	Ref.	Ref.	Ref.	Ref.	Ref.	Ref.
***Parental factors***						
**Mother's age**						
< 18	1.06 (0.49–2.31)	0.43 (0.20–0.89)^*^	1.63 (0.78–3.42)	1.12 (0.54–2.30)	1.76 (0.83–3.74)	1.11 (0.55–2.27)
18–24	0.98(0.71–1.37)	0.88 (0.63–1.23)	1.16 (0.91–1.48)	1.23 (0.93–1.61)	1.14 (0.81–1.60)	1.11 (0.78–1.58)
25–34	0.94 (0.71–1.25)	0.96 (0.72–1.28)	1.05 (0.88–1.25)	0.98 (0.81–1.20)	1.08 (0.81–1.43)	1.10 (0.82–1.49)
≥ 35	Ref.	Ref.	Ref.	Ref.	Ref.	Ref.
**Mother's education**						
No education	1.12 (0.97–1.29)	1.30 (1.12–1.50)^**^	1.32 (1.12–1.55)^*^	1.17 (0.98–1.41)	1.24 (1.07–1.44)^*^	1.37 (1.17–1.60)^**^
Primary and above	Ref.	Ref.	Ref.	Ref.	Ref.	Ref.
**Mother's occupation**						
Not working	Ref.	Ref.	Ref.	Ref.	Ref.	Ref.
Non-agriculture	0.94 (0.81–1.09)	1.06 (0.91–1.23)	0.89 (0.75–1.06)	0.82 (0.67–1.00)	0.87 (0.74–1.03)	0.91 (0.77–1.07)
Agriculture	0.99 (0.86–1.16)	1.33 (1.14–1.55)^**^	0.91 (0.77–1.08)	1.07 (0.88–1.28)	1.02 (0.87–1.18)	1.12 (0.96–1.32)
**Maternal stature**						
Normal/Tall	Ref.	Ref.	Ref.	Ref.	Ref.	Ref.
Short	1.98 (1.75–2.24)^**^	1.71 (1.51–1.93)^**^	1.02 (0.89–1.16)	0.91 (0.78–1.06)	1.81 (1.59–2.04)^**^	1.43 (1.25–1.62)^**^
Very short	3.30 (2.21–4.92)^**^	2.59 (1.77–3.80)^**^	1.13 (0.73–1.73)	0.90 (0.55–1.47)	2.49 (1.71–3.64)^**^	2.21 (1.52–3.22)^**^
**Listening to radio**						
Yes	Ref.	Ref.	Ref.	Ref.	Ref.	Ref.
Not at all	1.03 (0.90–1.18)	1.01 (0.89–1.14)	1.03 (0.89–1.20)	1.26 (1.06–1.50)*	1.07 (0.93–1.23)	0.91 (0.78–1.05)
**Watching television**						
Yes	Ref.	Ref.	Ref.	Ref.	Ref.	Ref.
Not at all	1.13 (0.95–1.34)	1.15 (0.96–1.38)	1.37 (1.12–1.68)^*^	1.47 (1.16–1.87)*	1.21 (1.01–1.45)^*^	1.30 (1.07–1.58)^*^
***Household factors***						
**Wealth index**						
Poor	Ref.	Ref.	Ref.	Ref.	Ref.	Ref.
Middle	0.93 (0.79–1.10)	0.73 (0.62–0.87)^**^	0.74 (0.61–0.88)^*^	0.94 (0.77–1.14)	0.75 (0.63–0.89)^*^	0.77 (0.64–0.92)^*^
Rich	0.91 (0.78–1.07)	0.76 (0.64–0.90)^*^	0.62 (0.51–0.75)^*^	0.71 (0.57–0.87)*	0.68 (0.58–0.81)^**^	0.63 (0.53–0.75)^**^
**Household size**						
1–4	0.93 (0.81–1.08)	0.93 (0.80–1.09)	0.96 (0.81–1.14)	0.82 (0.68–1.01)	0.84 (0.72–0.98)^*^	1.01 (0.86–1.19)
≥ 5	Ref.	Ref.	Ref.	Ref.	Ref.	Ref.
**Toilet facility**						
Improved	Ref.	Ref.	Ref.	Ref.	Ref.	Ref.
Unimproved	1.35 (1.11–1.63)^*^	1.32 (1.08–1.62)^*^	1.07 (0.85–1.34)	1.33 (1.01–1.74)^*^	1.63 (1.32–2.01)^**^	1.36 (1.08–1.72)^*^
**Source of drinking water**						
Improved	Ref	Ref	Ref	Ref.	Ref.	Ref.
Unimproved	1.08 (0.94–1.23)	0.88 (0.77–1.02)	0.98 (0.84–1.14)	0.80 (0.67–0.95)*	0.97 (0.84–1.12)	0.90 (0.78–1.05)
**Time to get a water source**						
On-premise	Ref.	Ref.	Ref.	Ref.	Ref.	Ref.
≤ 30 min	0.98 (0.77–1.26)	1.30 (0.99–1.69)	1.03 (0.77–1.38)	1.02 (0.72–1.42)	0.83 (0.63–1.08)	1.31 (0.96–1.77)
31–60 min	1.05 (0.80–1.37)	1.19 (0.89–1.60)	1.08 (0.79–1.48)	1.09 (0.76–1.57)	0.85 (0.63–1.14)	1.13 (0.81–1.57)
> 60 min	1.01 (0.77–1.33)	1.27 (0.95–1.72)	1.29 (0.94–1.76)	1.30 (0.90–1.87)	0.87 (0.65–1.18)	1.45 (1.04–2.02)*
***Community-level characteristics***						
**Residence**						
Urban	0.67 (0.53–0.84)^*^	0.77 (0.60–0.98)^*^	1.57 (1.21–2.04)^*^	1.41 (1.04–1.92)^*^	0.72 (0.56–0.93)^*^	1.03 (0.78–1.34)
Rural	Ref.	Ref.	Ref.	Ref.	Ref.	Ref.
**Region**						
Agrarian	Ref	Ref	Ref	Ref	Ref	Ref
Pastoralist	0.87 (0.76–1.01)	1.03 (0.89–1.20)	1.04 (0.90–1.22)	1.01 (0.85–1.20)	1.14 (0.98–1.32)	0.95 (0.81–1.10)
City administration	0.70 (0.57–0.86)^*^	0.84 (0.68–1.03)	0.98 (0.78–1.23)	0.97 (0.75–1.25)	0.98 (0.77–1.25)	0.83 (0.66–1.05)
**EDHS**						
2000	1.72 (1.64–1.89)^*^	1.51 (1.14–1.77)^*^	1.12 (0.74–1.29)	1.12 (0.74–1.29)	1.57 (1.43–1.72)*	1.33 (1.24–1.67)^*^
2005	1.68 (1.41–2.03)^*^	1.44 (1.19–1.73)^**^	1.09 (0.89–1.34)	0.94 (0.75–1.17)	1.41 (1.17–1.70)^**^	1.22 (1.05–1.49)^*^
2011	1.27 (1.10–1.45)^*^	1.15 (0.99–1.32)	1.03 (0.88–1.21)	0.91 (0.76–1.08)	1.39 (1.20–1.60)^**^	1.18 (1.02–1.38)^*^
2016	Ref	Ref	Ref	Ref	Ref	Ref
**Random effect**						
Variance (SE)	0.0971 (0.0031)^**^	0.0872 (0.0038)*	0.0877 (0.0045)^**^	0.1187 (0.0052)^*^	0.1073 (0.0034)^**^	0.0663(0.0052)^*^
ICC	2.86	2.58	2.59	3.48	3.15	1.97
**Model fitness**						
AIC	7530.55	7105.74	6988.41	5856.96	7248.70	6524.17
BIC	7795.59	7369.34	7251.12	6118.52	7507.34	6781.37

*EDHS* Ethiopia Demographic and Health Survey, *AIC* Akaike’s Information Criterion, *BIC* Bayesian information criterion, *ICC* Inter-cluster correlation

^*^p < 0.05

^**^p < 0.001

### Factors associated with stunting in male and female children

Table 5 presents a multilevel gender-specific disaggregated analysis for all outcome variables. Several factors were associated with childhood stunting in male and female children. At the individual level, the age of the child, perceived size of the child at birth, breastfeeding status, maternal stature, and type of the toilet facility at home were associated with stunting in both male and female children. At the community level, the place of residence (urban) showed a significant association with childhood stunting in both male and female children.

However, there are variations in the factor identified with childhood stunting among boys and girls. Unlike male children, for female children, mother's age, education level of the mother, mother's occupation and household wealth index, were determinants of childhood stunting. Female children born of mothers with no education (AOR: 1.30, 95%CI: 1.12–1.50) and working in agriculture (AOR: 1.33, 95%CI: 1.14–1.55) had higher odds of stunting. Among female children, the odds of stunting were lower among children from a household with a middle (AOR: 0.73, 95%CI: 0.62–0.87) and rich wealth index (AOR: 0.76, 95%CI: 0.64–0.90). On the other hand, unlike girls, boys living in cities had lower odds of being stunted (AOR: 0.70, 95%CI: 0.57–0.86) (Table 5).

### Factors associated with wasting in male and female children

Both individual and community-level factors were associated with wasting in both male and female children. The child's age (except those in the age category of 24–35 months), the perceived size of the child at birth (small birth size), the presence of diarrhea and fever, the mother's education, maternal stature, wealth index (rich households), and place of residence were identified factors associated with wasting in boys and girls (Table 5).

Unlike the girls, the odds of wasting were higher (AOR: 1.29, 95%CI: 1.06–1.58), for male children aged 24–35 months compared to children aged 36–59 months. There were higher odds of wasting among male children who were perceived by their mothers to be average sized at birth (AOR: 1.27, 95% CI: 1.09–1.49). Male children born to mothers with no education (AOR: 1.32, 95%CI: 1.12–1.55) were associated with higher odds of wasting. Children from a household with a middle wealth status (AOR: 0.74, 95%CI: 0.61–0.88) were associated with reduced odds of wasting (Table 5).

In female children, the child's age, the perceived size of the child at birth, diarrhea, fever in the last two weeks prior to the survey, household wealth index, and residence were similar determinants of wasting to that of male children. However, unlike boys, the odds of wasting were significantly higher among female children not fully vaccinated (AOR: 1.24, 95%: 1.02–1.52). Female children in a household with an unimproved toilet facility (AOR: 1.33, 95%CI: 1.01–1.74) were associated with increased odds of wasting. However, we observed an inverse association between unimproved sources of drinking water and childhood wasting (AOR: 0.80, 95%CI: 0.67–0.95) (Table 5).

### Factors associated with underweight in male and female children

The child's age, size of child at birth, diarrhea, and fever in the last two weeks, mother's education status, maternal stature, household wealth index, and toilet facility were factors associated with being underweight in both male and female children. Among male children, the odds of being underweight were lower among firstborn children (AOR: 0.77, 95%CI: 0.59–0.98), children living with 1–4 household size (AOR: 0.84, 95%CI: 0.72–0.98), and those from urban setting (AOR: 0.72, 95%CI: 0.56–0.93). On the other hand, among female children, the odds of being underweight were higher among children who were not fully vaccinated (AOR: 1.21, 95%CI: 1.03–1.41), had a fever in the last two weeks prior to the survey, and children born in households whose water source was away from the house for more than 60 min round trip (AOR: 1.45, 95%CI: 1.04–2.02) (Table 5).

## Discussion

Despite the significant impact of childhood undernutrition on child survival and development in Ethiopia, few studies have focused on gender inequality and identification of associated risk factors. Although a limited number of studies have been conducted in Ethiopia on childhood stunting, wasting and underweight among Ethiopian children [19, 21, 23, 29, 55–58], investigation of the burden of undernutrition focusing on gender inequality has not been adequately addressed in scientific papers [37, 59, 60] and efforts to reduce this inequality are rarely seen. Moreover, inequality studies primary focus on socioeconomic inequality [32–34, 37]. To our knowledge, our study is the first of its kind to assess the prevalence and factors associated with childhood undernutrition between boys and girls, using a nationally representative sample of children aged between 0 and 59 months in Ethiopia. Our findings showed that in Ethiopian, there are gender differences in childhood undernutrition in under-five children, with boys being more likely than girls to be stunted, wasted, and underweight. The common significant factors for childhood stunting in male and female children were the child's age, the perceived size of the child at birth, breastfeeding status, maternal stature, type of toilet facility, and place of residence. However, when comparing boys and girls, there are differences in risk factors for stunting, wasting, and underweight. Among female children, the education level of the mother, the mother's occupation, and the household wealth index were further identified as factors associated with childhood stunting. Unlike girls, the odds of wasting was higher for male children who were perceived by their mothers to be average sized at birth and children born to mothers with no education. Additionally, among male children, the odds of being underweight were lower among firstborn children, children living in 1–4 household size, and those from urban setting.

In this study, through all age groups (i.e., < 6 months, 6–11, 12–23, 24–35, and 36–59 months) and across all survey years male children had a higher prevalence of stunting, wasting and underweight compared to girls. The present analysis provides the evidence that inform the role that gender plays in childhood undernutrition in Ethiopia, highlighting the necessity of sex-specific nutritional interventions for promoting optimal child growth and development in the country. The high prevalence of undernutrition among boys observed in the current analyses is consistent with previous findings from Ethiopia [12, 14, 61, 62]. This finding is also consistent with the most recent systematic review and meta-analysis, which found that boys had a higher risk of stunting, wasting, and being underweight than girls [30]. Additionally, pooled evidence of studies conducted from 2008 to 2020 from 35 sub-Saharan African (SSA) countries reported that being a male child was associated with higher odds of stunting [63]. The higher likelihood of male children being wasted in this study mirrors the previous findings in India and Ghana [64, 65]. Similarly, prior studies in Ethiopia [62], and different low-income settings such as Kenya [66], Rwanda [67], Senegal [68], Tanzania [69] and Indonesia [70] have reported that being underweight was the most common undernutrition problem among boys than girls. Our study extends the body of knowledge including informing the need to put significant emphasis of targeting a male child in addressing undernutrition, thus addressing gender inequality As well, given that the previous studies in Ethiopia have had one of the following limitations: either dated (e.g. survey specific and based on the 2011 EDHS [37]), consisted of a narrower age band, socioeconomic status or geographically specific [36], they limited their generalisability at the national level.

Studies investigating the role of gender on child development are still limited in Ethiopia. However, many questions remain unanswered, such as the causal direction of undernutrition and gender and the possible mechanism that explains this interaction in children aged under five years. Several possible explanations have been reported concerning gender differences in undernutrition status. According to Thompson (2021) [71], boys may be more vulnerable to illness and malnourishment due to sex differences in immune system development. Sex-based biological factors may also make boys more vulnerable to infection [30, 71]. There is also evidence that, men are more susceptible to infections than women [72–74], and that boys are more likely to be born preterm, increasing their risk for most adverse neonatal outcomes [75] and a higher risk of perinatal complications [76]. Furthermore, studies show that boys have a shorter median duration of predominant breastfeeding than girls [45], and there are differences in growth and immune function between these two genders [77]. The roles that boys and girls play in a community, as well as the values associated with them, may have an impact on their nutritional status [78]. Identifying the various causes, mechanisms, and effects of sex variations in undernutrition necessitates additional research to discover whether sex differences affect child growth, development and long-term morbidity and mortality rates.

A child's age is a common determinant of stunting for both boys and girls. The odds of stunting were lower among young children (i.e., aged 0–23 months) than those in the age category of 36–59 months in both sexes, consistent with several other related studies conducted elsewhere [56, 79–83]. The observed lower odds of stunting among children aged 0 to 23 months could be explained by the protective effect of breastfeeding, as most Ethiopian children are breastfed until the age of two years [84]. Additionally, in countries like Ethiopia where food security is still a serious issue, a lack of adequate and balanced food intake to meet the metabolic demand of older children leaves a child vulnerable to a high risk of stunting. Further studies could examine which age groups, genders, and why they do suffer from undernutrition.

Our study revealed that the likelihood of stunting was higher among children perceived by their mothers as smaller and average size than normal at birth among boys and girls. In agreement with the current study, previous studies in India, Bangladesh, and Nepal have also stated that children born with smaller than average size were more prone to stunting [85–87]. We speculate that the observed results may be due to children's low birth weight, which may have an effect on child liner growth. In our study, having a very short and short mother was associated with increased odds of stunting among children of both sexes. Stunting is related to maternal stature [88–90]. This finding evidenced that stunting is passed from one generation to the next in an intergenerational cycle, indicating that children born to stunted mothers are more likely to be stunted themselves [4, 91].

The relationship between unimproved water, sanitation and hygiene (WASH) and child undernutrition has been widely acknowledged by previous literature [22, 92–94]. Not surprisingly, in the current study, children in households with inadequate toilet facilities were at a higher risk of stunting. The findings suggest that children living in households with poor toilet facilities or in areas where open defecation was widespread were more vulnerable to stunting, which could be attributed to frequent episodes of diarrheal disease. Diarrhea contributes to nutritional deficiencies by reducing food intake, decreasing nutrient absorption, and promoting nutrient catabolism. There is a clear biological mechanism and plausibility for the impact of WASH access on nutritional status. WASH is dependent not just on the availability of factors including water and sanitation facilities, but also on proper health behaviors (e.g., hand washing at crucial times) by all or key members of the family (e.g., caregiver cleaning a child's hands). Hence, attention to this issue is of particular importance, as large interventional studies, such as the WASH-Benefits Bangladesh [95], the WASH-Benefits Kenya [96] and the Sanitation Hygiene Infant Nutrition Efficacy (SHINE) trials in Zimbabwe [97] have found no effects of any WASH intervention on child linear growth. These findings suggest that poor WASH affects children’s developmental processes in complex ways, spanning multiple routes.

Among the girl children, our study indicates that children born to mothers with no education and working in agriculture were more likely to be stunted. The growing body of evidence connects maternal employment to children's nutritional status. For example, one study from the nine Demographic and Health Surveys conducted in various countries (Benin, Burundi, Cambodia, Congo, Haiti, Rwanda, Senegal, Togo, and Uganda) found that parental agricultural employment, relative to non-agricultural employment, was associated with poorer child development [98]. Further studies could further explore why certain maternal employment influenced children's nutritional status in different sexes.

Similar to previous studies [99, 100], a significant association between mothers' education and stunting was found. This result is more predictable because education gives mothers the information necessary to be knowledgeable about child nutrition, hygiene, and health. That is, a mother with a higher level of education is associated with better childcare practices. Studies have also found that knowledge of these things strongly predicts stunting in children under five years [101].

This finding reaffirms that children from relatively wealthiest households and living in an urban setting were less likely to be stunted compared to their counterparts. This association may reflect that greater wealth ensures a sufficient nutritionally balanced household food supply and a better living environment, and a variety of other essential characteristics for effective child growth and development. Additionally, previous studies in Ethiopia [14, 15], Rwanda [102], Ghana [103], and Tanzania [104] found a link between higher wealth status and a lower risk of undernutrition.

In our analyses, for both boys and girls of younger age, children who were perceived by their mothers to be smaller than normal at birth, children who had diarrhea and fever in the last two weeks prior to the survey and living in urban residences were significant independent risk factors of childhood wasting. Our findings also indicate that children from relatively wealthiest households had lower odds of wasting than children from poor households for both sexes. We hypothesize that children from richer households afford foods high in nutritional value, have lower intestinal infections and live in food secure households. Corresponding with our findings, the World Health Organizations Framework for Social Determinants of Health (SDH) acknowledged that poverty, low level of education, the environment where people live and work, determine population health outcomes [105].

Unlike girls, boys born to mothers with no education had higher odds of being wasted. On the other hand, those living in households with unimproved toilet facilities was one of the factors associated with wasting among girls. These results corroborate other studies, where children who had diarrhea [28], reported to have been born with low birth size [106–108], living in urban areas [27], and had poor socioeconomic status [28, 109] were more likely to be wasted.

Several factors were associated with child underweight in both boys and girls. The most consistent factors identified were younger child age, the perceived size of the child at birth, diarrhea, maternal education, maternal stature, watching television, wealth index, and household sanitation facility. However, among boys, birth order, household size, and place of residence were important factors significantly associated with being underweight. On the other hand, vaccination status, having fever, and time taken for a round trip to water sources were the identified factors associated with being underweight for girls.

The odds of being underweight were higher among children perceived as small size at birth. This is similar to various reports in Ethiopia and elsewhere [29, 64, 69, 110], confirming that birth size was an important determinant factor that significantly affected the nutritional status. The lower odds ratios of underweight for children in age groups (< 6 months and 6 to 11 months) observed among boys and girls also supports other studies from Bangladesh [111]. Our findings are consistent with other research showing strong positive associations between underweight and diarrhea in the last two weeks [29, 112, 113], short-statured mothers [114, 115], mothers with no education [29, 69, 112], and children from households that used unimproved toilets [69, 116, 117]. Our study also emphasized that children under five years from relatively wealthiest households are at a lower risk of being underweight than children from poor households [29, 111]. Wealth status serves as a substitute for higher socioeconomic status that improves the ability of mothers to afford the cost of nutritious food and ensured household food security. Children’s from relatively wealthiest households were found at lower risk of being underweight. The other finding of this study is birth order and place of residence, which had a strong positive association with being underweight. In agreement with related studies, boys' probability of being underweight for a firstborn child [29] and those residing in an urban setting [118] was lower.

### Limitations

The study has certain limitations. First, since this study was based on cross-sectional data, it could not provide evidence of a causal relationship between outcome and independent variables. Second, due to the unavailability of data on potential confounders, including household food security, the behavior of the parents, and underlying disease conditions, these were not included in the analysis. Third, some data on personal and household practices were based on the mothers’ recall, which might have been subjected to recall bias. Fourth, the pooling of the data may be affected by heterogeneity across survey years. Despite these limitations, the data were collected from across the country, making it a nationally representative study. In addition, using a nationwide population-based dataset provides a large sample size and statistical power to study identify factors for childhood stunting, wasting and underweight among under-five boys and girls in Ethiopia. Additionally, having used the national survey dataset from across multiple years when the four consecutive Ethiopia Demographic and Health Surveys were conducted (2000 to 2016), our findings provide a nationwide assessment of gender differentials in childhood undernutrition, and compares the prevalence of different forms of this significant health problem across the years, contributing evidence from the context of the whole of Ethiopia and over time. Further investigation is needed to understand how gender-sensitive interventions targeting child growth are successful and economically feasible, and we are aware that such studies are scarce in low-income countries such as Ethiopia.

## Conclusions

Our analyses show that childhood stunting, wasting, and being underweight were higher among boys than girls. The most consistent factors for childhood undernutrition for male and female children were the child's age, the perceived size of the child at birth, breastfeeding status, maternal stature, maternal education, toilet facility, wealth index, and place of residence. However, among female children, education level, the mother's occupation, and the household wealth index were factors identified to be associated with childhood stunting. Unlike girls, boys born to mothers with no education had higher odds of being wasted. On the other hand, those living in households with unimproved toilet facilities were more likely to have wasting among girls. Among boys, birth order and place of residence were important factors, significantly associated with being underweight. Moreover, vaccination status, fever, and time taken to get to and from the water sources were the factors identified to be associated with being underweight for girls. Overall, child sex has been identified as an important risk factor for childhood undernutrition in Ethiopia. Recently the government of Ethiopia endorsed 2019 Food and Nutrition Policy which aims to achieve optimal nutritional status throughout the life cycle via implementation of nutrition-specific and nutrition-sensitive interventions. The current finding will benefit and inform this policy as it informs the need to emphasize on gender-sensitive interventions to optimize infant and young child nutritional status. Furthermore, the Seqota Declaration, in which Ethiopia has made commitment to ending childhood undernutrition by 2030, should emphasize gender-sensitive programs to accelerate reductions in childhood undernutrition.

### Supplementary Information

**Additional file 1.**

**Additional file 2.**

**Additional file 3.**

**Additional file 4.**

**Additional file 5.**

**Additional file 6.**

## Data Availability

Data are available in a public, open access repository. Data for this study were sourced from Demographic and Health surveys (DHS) and available here: http://dhsprogram.com/data/available-datasets.cfm.
