# Supplementary material for: Gender-specific disaggregated analysis of childhood undernutrition in Ethiopia: evidence from 2000–2016 nationwide survey
Source: BMC Public Health. 2023 Oct 19;23:2040. doi: 10.1186/s12889-023-16907-x (PMC10585928; doi:10.1186/s12889-023-16907-x)
Supplement: Supplementary file 1 — Additional file 1. [file 12889_2023_16907_MOESM1_ESM.docx]

**Supplementary File 1: Variable definitions for some of the independent variables**

| **Variables** | **Variable type and category** | **Measurement/Definition** |
| --- | --- | --- |
| Size of the child at birth | Categorical data, categorized as “Large”, “Average” and “Small”. | In the EDHS survey, mothers were asked to estimate the size of their child based on their experience or by comparison with a previous child. Mothers’ perceived baby size at birth response options were ‘greater than average’, ‘average’, and ‘smaller than average ’and ‘very small’. This variable was recoded as “Large”, “Average” or “Small”, which is below average, smaller than average or very small. |
| Currently breastfeeding | Binary data, categorized as “Yes” or “No” | Categorized as “Yes” if the child is currently breastfeeding “No” otherwise. |
| Early initiation of breastfeeding | Binary data, categorized as “Yes” or “No” | Children born in the past 2 years who started breastfeeding within one hour of birth was coded as “Yes” or “No” |
| Had diarrhea in the last 2 weeks | Binary data, categorized as “Yes” or “No” | Mothers were asked if their children had diarrhea in the past 2 weeks prior to the survey. The response was recorded as “yes” and “no.” |
| Had fever in the last 2 weeks | Binary data, categorized as “Yes” or “No” | Mothers were asked if their children had fever and children with fever at any time in the 2 weeks preceding the survey were recorded as “yes” if not “no” |
| Full vaccination | Binary data, categorized as “Yes” or “No” | “Yes” if the child received a Bacillus Calmette–Guérin (BCG) vaccination against tuberculosis; 3 doses of Diptheria, pertussis, and tetanus vaccine (DPT); ≥3 doses of polio vaccine (OPV); and 1 dose of measles vaccine; no otherwise. |
| Mother's age | Categorical data, categorized as “<18”, “18-24”, “25-34”, or “>=35” | The self-reported age of the mother (in years) was categorized into four categories as “<18”, “18-24”, “25-34”, and “≥ 35”. |
| Maternal stature | Categorical data, categorized as (normal/tall, short, or very short) | Maternal stature was measured and categorized maternal height as: very short (< 145.0 cm), short (145.0 to 154.9 cm) and normal/tall (≥155.0 cm) |
| Mother's occupation | Categorical data, categorized as “Not working”, “Non agriculture”, or “Agriculture” | The survey asked women about their occupational status the responses were numerous. For this analysis, we re-coded the mother's occupation status into three categories for adequate sampling as “Not working”, “Non agriculture”, or “Agriculture” |
| Household Wealth index | Categorical data, categorized as “Poor”, “Middle”, or “Rich” | The household wealth index was calculated based on household assets, such as televisions and bicycles. Principal components analysis was applied to generate the wealth index as a continuous scale of relative wealth. The wealth index was categorized into five wealth quintiles: 'very poor', 'poor', 'middle', 'rich' and 'very rich. For this analysis, we re-coded the wealth index into three categories for adequate sampling in each category: 'poor' (poor and very poor), 'middle' and 'rich' (rich and very rich). |
| Toilet facility | Categorical data, categorized as “Improved", "Unimproved" or "Open defecation." | Based on the WHO definition, facilities would be considered improved if any of the following occured: flush/pour flush toilets to piped sewer systems, septic tanks, and pit latrines; ventilated improved pit (VIP) latrines; pit latrines with slabs; and composting toilets. Unimproved sanitation included: flush or pour-flush to elsewhere; pit latrine without a slab or open pit; bucket, hanging toilet or hanging latrine. Other facilities, including households with no facility or use of bush/field, were considered as open defecation. |
| Source of drinking water | Categorical data, categorized as “Improved”, or “Unimproved” | Improved sources of drinking water included piped water, public taps, standpipes, tube wells, boreholes, protected dug wells and springs, and rainwater. Other sources of drinking water were regarded as unimproved. |
| Region of residence | Categorical data, “Agrarian”, “Pastoralist”, or “City Administrations” | The variable was recoded as ‘agrarian’ (encompassing Tigray, Amhara, Oromia, Benishangul, SNNPR, and Gambela regions), ‘pastoralist’ (Afar and Somali regions), or ‘city dweller’ (Addis Ababa, Dire Dawa cites, and Harari regions). An agrarian society is any community whose economy is based on producing and maintaining crops and farmland. A pastoralist society is any community whose economy is based on raising livestock. A city-dweller society is any city community. |

kg, weight measured in kilograms; m^2^ height measured in square meters
