# Supplementary material for: Gender-specific disaggregated analysis of childhood undernutrition in Ethiopia: evidence from 2000–2016 nationwide survey
Source: BMC Public Health. 2023 Oct 19;23:2040. doi: 10.1186/s12889-023-16907-x (PMC10585928; doi:10.1186/s12889-023-16907-x)
Supplement: Supplementary file 2 — Additional file 2. [file 12889_2023_16907_MOESM2_ESM.docx]

**Supplementary File 2: Prevalence of undernutrition among boys and girls in children 0–59 months, EDHS 2000-2016**

| **Undernutrition status** | **EDHS-2000** | **95%CI** | **EDHS-2005** | **95%CI** | **EDHS-2011** | **95%CI** | **EDHS-2016** | **95%CI** | **Pooled (2000-2016)** | **95%CI** |
| --- | --- | --- | --- | --- | --- | --- | --- | --- | --- | --- |
| **Overall Stunting** | 57.4 | 56.5-58.4 | 50.9 | 49.4-52.4 | 44.3 | 43.3-45.3 | 38.4 | 37.4-39.3 | 47.3 | 46.8-47.8 |
| Stunting (Male) | 59.0 | 57.6-60.4 | 52.4 | 50.2-54.5 | 45.9 | 44.6-47.3 | 41.0 | 39.7-42.4 | 49.2 | 48.4-49.9 |
| Stunting (Female) | 55.8 | 54.4-57.2 | 49.4 | 47.3-51.5 | 42.5 | 41.1-43.9 | 35.6 | 34.2-36.9 | 45.4 | 44.6-46.1 |
| **Overall Wasting** | 12.5 | 11.8-13.1 | 12.2 | 11.3-13.2 | 9.7 | 9.2-10.3 | 10.1 | 9.5-10.7 | 10.9 | 10.6-11.3 |
| Wasting (Male) | 13.8 | 12.8-14.8 | 13.5 | 12.1-14.9 | 11.1 | 10.2-11.9 | 10.3 | 9.5-11.2 | 11.9 | 11.4-12.4 |
| Wasting (Female) | 11.1 | 10.3-12.1 | 10.9 | 9.7-12.2 | 8.3 | 7.6-9.2 | 9.8 | 8.9-10.7 | 9.9 | 9.5-10.4 |
| **Overall Underweight** | 41.1 | 40.1-42.1 | 33.2 | 31.8-34.7 | 28.8 | 27.9-29.7 | 23.8 | 22.9-24.6 | 31.5 | 31.0-32.0 |
| Underweight (Male) | 43.3 | 41.9-44.7 | 34.1 | 32.2-36.2 | 30.5 | 29.2-31.7 | 25.3 | 24.1-26.5 | 33.1 | 32.4-33.8 |
| Underweight (Female) | 38.8 | 37.5-40.2 | 32.3 | 30.4-34.4 | 27.1 | 25.8-28.3 | 22.2 | 21.1-23.4 | 29.8 | 29.1-30.5 |
