# Supplementary figures and images for: Gender-specific disaggregated analysis of childhood undernutrition in Ethiopia: evidence from 2000–2016 nationwide survey
Source: BMC Public Health. 2023 Oct 19;23:2040. doi: 10.1186/s12889-023-16907-x (PMC10585928; doi:10.1186/s12889-023-16907-x)

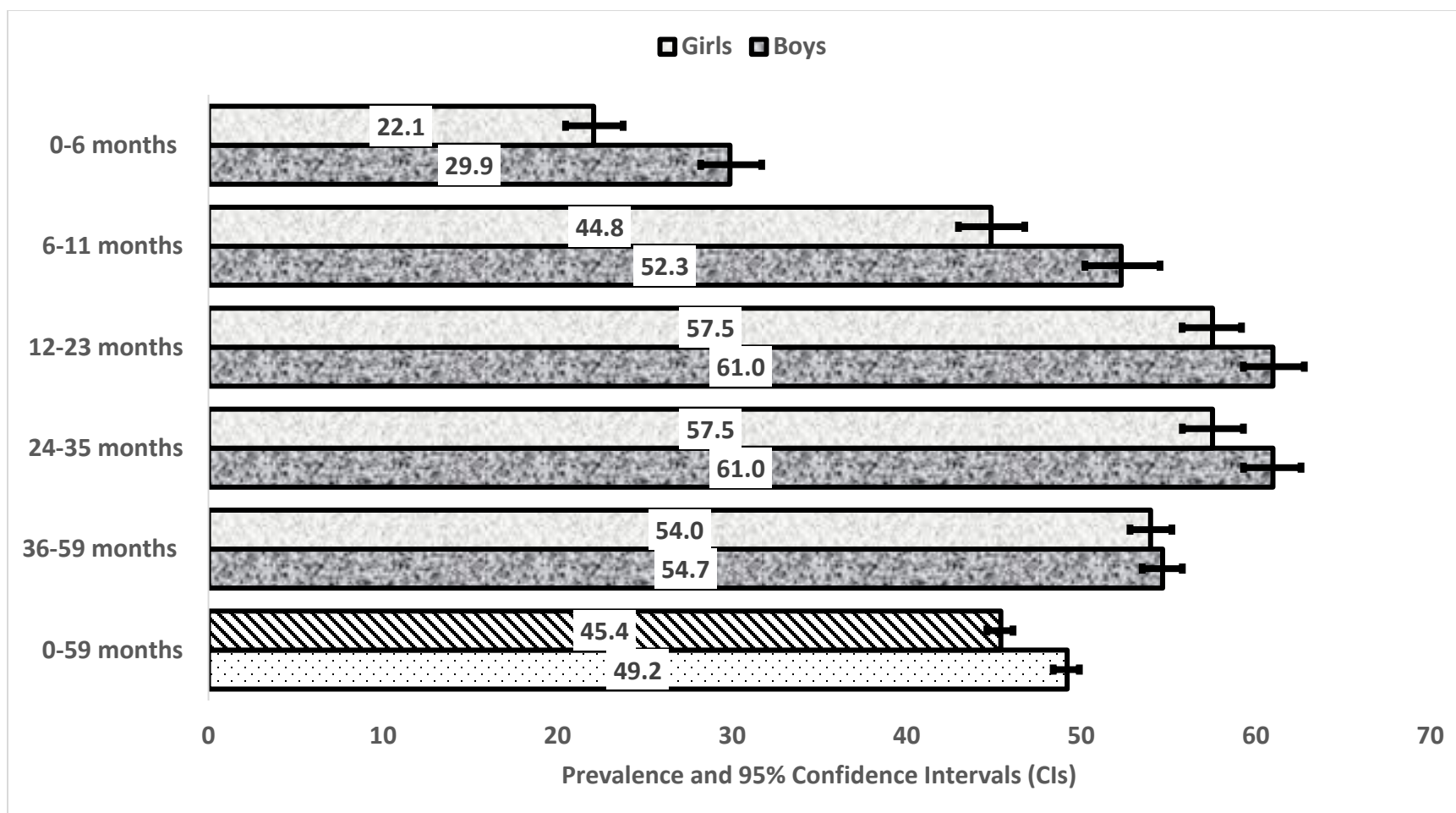

**Supplementary File 3: Stunting in different EDHS survey years, by age in Ethiopia**

Supplement: Supplementary file 3 — Additional file 3. [file 12889_2023_16907_MOESM3_ESM.pdf]

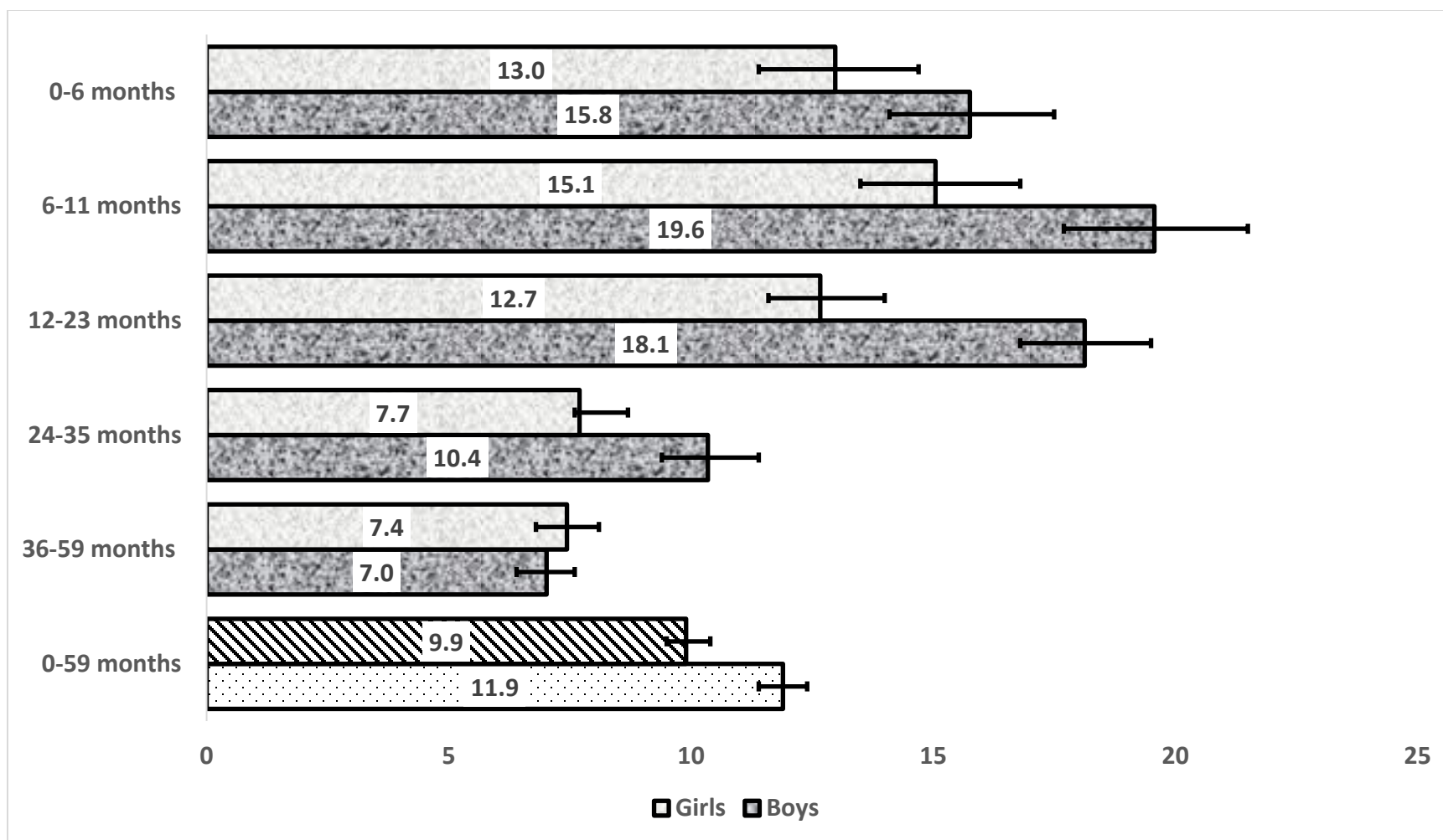

**Supplementary File 4: Wasting in different EDHS survey years, by age in Ethiopia**

Supplement: Supplementary file 4 — Additional file 4. [file 12889_2023_16907_MOESM4_ESM.pdf]

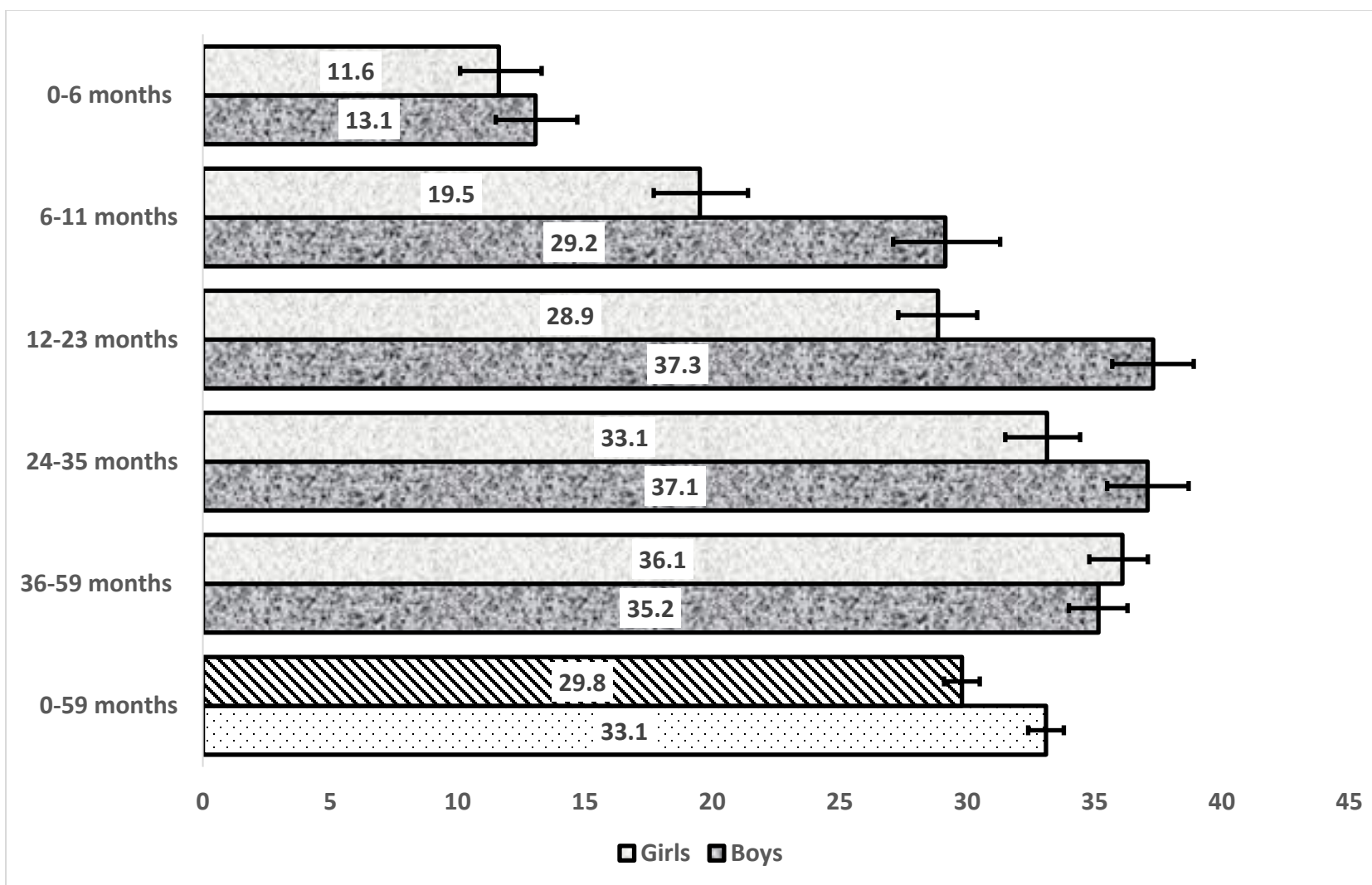

**Supplementary File 5: Underweight in different EDHS survey years, by age in Ethiopia**

Supplement: Supplementary file 5 — Additional file 5. [file 12889_2023_16907_MOESM5_ESM.pdf]
