# Supplementary material for: Gender-specific disaggregated analysis of childhood undernutrition in Ethiopia: evidence from 2000–2016 nationwide survey
Source: BMC Public Health. 2023 Oct 19;23:2040. doi: 10.1186/s12889-023-16907-x (PMC10585928; doi:10.1186/s12889-023-16907-x)
Supplement: Supplementary file 6 — Additional file 6. [file 12889_2023_16907_MOESM6_ESM.docx]

**Supplementary File 6: Multivariable multilevel models on factors associated with stunting, wasting, and underweight among boys and girls in children 0–59 months, EDHS 2000-2016**

| Variables | **Stunting** | | | **Wasting** | | | **Underweight** | | |
| --- | --- | --- | --- | --- | --- | --- | --- | --- | --- |
|  | Male | Female | Overall (Boys & Girls) | Male | Female | Overall (Boys & Girls) | Male | Female | Overall (Boys & Girls) |
|  | AOR (95%CI%) | AOR (95%CI%) | AOR (95%CI%) | AOR (95%CI%) | AOR (95%CI%) | AOR (95%CI%) | AOR (95%CI%) | AOR (95%CI%) | AOR (95%CI%) |
| ***Child factors*** |  |  |  |  |  |  |  |  |  |
| **Sex** |  |  |  |  |  |  |  |  |  |
| Male |  |  | ***1.31 (1.21-1.42)***** |  |  | ***1.35 (1.23-1.48)***** |  |  | ***1.38 (1.26-1.50)***** |
| Female |  |  | Ref. |  |  | Ref. |  |  | Ref. |
| **Age (months)** |  |  |  |  |  |  |  |  |  |
| <6 | 0.06 (0.05-0.08)** | 0.08 (0.06-0.11)** | 0.07 (0.06-0.0)** | 2.12 (1.68-2.68)** | 1.77 (1.37-2.29)** | 1.95 (1.64-2.32)** | 0.21 (0.16-0.26)** | 0.17 (0.13-0.22)** | 0.19 (0.15-0.22)** |
| 6-11 | 0.14 (0.11-0.18)** | 0.12 (0.09-0.16)** | 0.13 (0.11-0.16)** | 2.37 (1.88-2.97)** | 1.84 (1.44-2.36)** | 2.11 (1.78-2.49)** | 0.49 (0.39-0.62)** | 0.30 (0.23-0.37)** | 0.39 (0.33-0.45)** |
| 12-23 | 0.54 (0.43-0.66)** | 0.47 (0.38-0.58)** | 0.50 (0.43-0.58)** | 2.08 (1.71-2.54)** | 1.66 (1.34-2.06)** | 1.87 (1.61-2.16)** | 0.88 (0.73-1.06) | 0.61 (0.50-0.73)** | 0.73 (0.64-0.83)** |
| 24-35 | 1.13 (0.93-1.36) | 1.14 (0.95-1.38) | 1.14 (0.93-1.29) | 1.29 (1.06-1.58)* | 0.93 (0.74-1.17) | 1.12 (0.96-1.30) | 1.18 (0.98-1.43) | 0.91 (0.75-1.09) | 1.03 (0.90-1.18) |
| 36-59 | Ref. | Ref. | Ref. | Ref. | Ref. | Ref. | Ref. | Ref. | Ref. |
| **Birth order** |  |  |  |  |  |  |  |  |  |
| First born | 0.98 (0.77-1.25) | 1.20 (0.94-1.54) | 1.09 (0.92-1.29) | 0.78 (0.60-1.01) | 0.85 (0.63-1.13) | 0.81 (0.67-0.98)* | 0.77 (0.59-0.98)* | 0.82 (0.63-1.07) | 0.79 (0.66-0.95)** |
| 2-4 | 0.88 (0.75-1.03) | 1.04 (0.88-1.22) | 0.97 (0.86-1.08) | 0.89 (0.75-1.06) | 0.95 (0.79-1.14) | 0.91 (0.81-1.03) | 0.85 (0.72-1.01) | 0.93 (0.78-1.11) | 0.89 (0.79-1.01) |
| 5 or higher | Ref. | Ref. | Ref. | Ref. | Ref. | Ref. | Ref. | Ref. | Ref. |
| **Birth interval** |  |  |  |  |  |  |  |  |  |
| < 33 months | Ref. | Ref. | Ref. | - | - | - | Ref. | Ref. | Ref. |
| ≥33 months | 0.87 (0.66-1.15) | 0.89 (0.68-1.18) | 0.88 (0.73-1.07) |  |  |  | 0.87 (0.66-1.16) | 1.03 (0.77-1.36) | 0.95 (0.77-1.15) |
| **Size of child at birth** |  |  |  |  |  |  |  |  |  |
| Larger | Ref. | Ref. | Ref. | Ref. | Ref. | Ref. | Ref. | Ref. | Ref. |
| Average | 1.31 (1.15-1.50)** | 1.17 (1.01-1.36)* | 1.24 (1.12-1.37)** | 1.27 (1.09-1.49)* | 1.03 (0.85-1.25) | 1.17 (1.04-1.32)* | 1.36 (1.18-1.57)** | 1.22 (1.04-1.44)* | 1.30 (1.16-1.45)** |
| Small | 1.80 (1.55-2.09)** | 1.57 (1.35-1.83)** | 1.68 (1.51-1.87)** | 1.73 (1.46-2.03)** | 1.55 (1.29-1.87)** | 1.65 (1.46-1.87)** | 2.46 (2.11-2.89)** | 1.94 (1.65-2.29)** | 2.18 (2.95-2.44)** |
| **Currently breastfeeding** |  |  |  |  |  |  |  |  |  |
| Yes | Ref. | Ref. | Ref. | Ref. | Ref. | Ref. | - | - | - |
| No | 0.66 (0.56-0.78)** | 0.71 (0.60-0.83)** | 0.68 (0.61-0.77)** | 1.03 (0.87-1.21) | 0.99 (0.82-1.19) | 1.01 (0.89-1.14) |  |  |  |
| **Early initiation of breastfeeding** |  |  |  |  |  |  |  |  |  |
| Yes | Ref. | Ref. | Ref. | - | - | - | Ref. | Ref. | Ref. |
| No | 1.02 (0.90-1.14) | 1.01 (0.89-1.14) | 1.02 (0.93-1.11) |  |  |  | 0.93 (0.82-1.05) | 1.01 (0.88-1.14) | 0.96 (0.88-1.05) |
| **Full vaccination** |  |  |  |  |  |  |  |  |  |
| Yes | Ref. | Ref. | Ref. | Ref. | Ref. | Ref. | Ref. | Ref. | Ref. |
| No | 1.04 (0.90-1.19) | 1.01 (0.88-1.17) | 1.03 (0.93-1.14) | 1.09 (0.92-1.29) | 1.24 (1.02-1.52)* | 1.15 (1.01-1.31)* | 1.06 (0.92-1.23) | 1.21 (1.03-1.41)* | 1.12 (1.01-1.25)* |
| **Diarrhea** |  |  |  |  |  |  |  |  |  |
| Yes | 1.16 (0.99-1.35) | 1.04 (0.88-1.22) | 1.09 (0.98-1.23) | 1.25 (1.06-1.48)* | 1.25 (1.03-1.51)* | 1.26 (1.12-1.43)** | 1.26 (1.08-1.47)* | 1.32 (1.12-1.56)* | 1.28 (1.15-1.44)** |
| No | Ref. | Ref. | Ref. | Ref. | Ref. | Ref. | Ref. | Ref. | Ref. |
| **Fever** |  |  |  |  |  |  |  |  |  |
| Yes | 0.99 (0.86-1.15) | 1.02 (0.87-1.19) | 1.02 (0.92-1.13) | 1.20 (1.02-1.41)* | 1.48 (1.25-1.77)** | 1.31 (1.16-1.47)** | 1.14 (0.99-1.33) | 1.18 (1.01-1.38)* | 1.16 (1.04-1.29)* |
| No | Ref. | Ref. | Ref. | Ref. | Ref. | Ref. | Ref. | Ref. | Ref. |
| ***Parental factors*** |  |  |  |  |  |  |  |  |  |
| **Mother's age** |  |  |  |  |  |  |  |  |  |
| <18 | 1.06 (0.49-2.31) | 0.43 (0.20-0.89)* | 0.63 (0.37-1.06) | 1.63 (0.78-3.42) | 1.12 (0.54-2.30) | 1.30 (0.78-2.17) | 1.76 (0.83-3.74) | 1.11 (0.55-2.27) | 1.31 (0.79-2.18) |
| 18-24 | 0.98(0.71-1.37) | 0.88 (0.63-1.23) | 0.92(0.73-1.17) | 1.16 (0.91-1.48) | 1.23 (0.93-1.61) | 1.19 (0.99-1.43) | 1.14 (0.81-1.60) | 1.11 (0.78-1.58) | 1.12 (0.88-1.42) |
| 25-34 | 0.94 (0.71-1.25) | 0.96 (0.72-1.28) | 0.95 (0.78-1.16) | 1.05 (0.88-1.25) | 0.98 (0.81-1.20) | 1.02 (0.89-1.16) | 1.08 (0.81-1.43) | 1.10 (0.82-1.49) | 1.08 (0.88-1.33) |
| ≥35 | Ref. | Ref. | Ref. | Ref. | Ref. | Ref. | Ref. | Ref. | Ref. |
| **Mother's education** |  |  |  |  |  |  |  |  |  |
| No education | 1.12 (0.97-1.29) | 1.30 (1.12-1.50)** | 1.20 (1.08-1.33)** | 1.32 (1.12-1.55)* | 1.17 (0.98-1.41) | 1.25 (1.11-1.41)** | 1.24 (1.07-1.44)* | 1.37 (1.17-1.60)** | 1.30 (1.17-1.45)** |
| Primary and above | Ref. | Ref. | Ref. | Ref. | Ref. | Ref. | Ref. | Ref. | Ref. |
| **Mother's occupation** |  |  |  |  |  |  |  |  |  |
| Not working | Ref. | Ref. | Ref. | Ref. | Ref. | Ref. | Ref. | Ref. | Ref. |
| Non agriculture | 0.94 (0.81-1.09) | 1.06 (0.91-1.23) | 0.99 (0.88-1.10) | 0.89 (0.75-1.06) | 0.82 (0.67-1.00) | 0.87 (0.77-0.99)* | 0.87 (0.74-1.03) | 0.91 (0.77-1.07) | 0.89 (0.79-0.99)* |
| Agriculture | 0.99 (0.86-1.16) | 1.33 (1.14-1.55)** | 1.13 (1.01-1.26)* | 0.91 (0.77-1.08) | 1.07 (0.88-1.28) | 0.99 (0.87-1.12) | 1.02 (0.87-1.18) | 1.12 (0.96-1.32) | 1.06 (0.94-1.18) |
| **Maternal stature** |  |  |  |  |  |  |  |  |  |
| Normal/Tall (>=155 cm) | Ref. | Ref. | Ref. | Ref. | Ref. | Ref. | Ref. | Ref. | Ref. |
| Short (145 to 154.9 cm) | 1.98 (1.75-2.24)** | 1.71 (1.51-1.93)** | 1.82 (1.67-1.98)** | 1.02 (0.89-1.16) | 0.91 (0.78-1.06) | 0.98 (0.88-1.08) | 1.81 (1.59-2.04)** | 1.43 (1.25-1.62)** | 1.81 (1.59-2.04)** |
| Very short (<145 cm) | 3.30 (2.21-4.92)** | 2.59 (1.77-3.80)** | 2.93 (2.23-3.86)** | 1.13 (0.73-1.73) | 0.90 (0.55-1.47) | 1.02 (0.74-1.41) | 2.49 (1.71-3.64)** | 2.21 (1.52-3.22)** | 2.49 (1.71-3.64)** |
| **Listening to radio** |  |  |  |  |  |  |  |  |  |
| Yes | Ref. | Ref. | Ref. | Ref. | Ref. | Ref. | Ref. | Ref. | Ref. |
| Not at all | 1.03 (0.90-1.18) | 1.01 (0.89-1.14) | 1.01 (0.92-1.12) | 1.03 (0.89-1.20) | 1.26 (1.06-1.50)* | 1.12 (1.01-1.25)* | 1.07 (0.93-1.23) | 0.91 (0.78-1.05) | 0.99 (0.89-1.09) |
| **Watching television** |  |  |  |  |  |  |  |  |  |
| Yes | Ref. | Ref. | Ref. | Ref. | Ref. | Ref. | Ref. | Ref. | Ref. |
| Not at all | 1.13 (0.95-1.34) | 1.15 (0.96-1.38) | 1.14 (1.01-1.28)* | 1.37 (1.12-1.68)* | 1.47 (1.16-1.87)* | 1.41 (1.21-1.65)** | 1.21 (1.01-1.45)* | 1.30 (1.07-1.58)* | 1.23 (1.08-1.41)* |
| ***Household factors*** |  |  |  |  |  |  |  |  |  |
| **Wealth index** |  |  |  |  |  |  |  |  |  |
| Poor | Ref. | Ref. | Ref. | Ref. | Ref. | Ref. | Ref. | Ref. | Ref. |
| Middle | 0.93 (0.79-1.10) | 0.73 (0.62-0.87)** | 0.84 (0.74-0.94)* | 0.74 (0.61-0.88)* | 0.94 (0.77-1.14) | 0.83 (0.72-0.95)* | 0.75 (0.63-0.89)* | 0.77 (0.64-0.92)* | 0.77 (0.68-0.87)** |
| Rich | 0.91 (0.78-1.07) | 0.76 (0.64-0.90)* | 0.83 (0.74-0.93)* | 0.62 (0.51-0.75)* | 0.71 (0.57-0.87)* | 0.66 (0.57-0.76)** | 0.68 (0.58-0.81)** | 0.63 (0.53-0.75)** | 0.66 (0.58-0.75)** |
| **Household size** |  |  |  |  |  |  |  |  |  |
| 1-4 | 0.93 (0.81-1.08) | 0.93 (0.80-1.09) | 0.93 (0.83-1.03) | 0.96 (0.81-1.14) | 0.82 (0.68-1.01) | 0.90 (0.79-1.02) | 0.84 (0.72-0.98)* | 1.01 (0.86-1.19) | 0.92 (0.82-1.03) |
| ≥ 5 | Ref. | Ref. | Ref. | Ref. | Ref. | Ref. | Ref. | Ref. | Ref. |
| **Toilet facility** |  |  |  |  |  |  |  |  |  |
| Improved | Ref. | Ref. | Ref. | Ref. | Ref. | Ref. | Ref. | Ref. | Ref. |
| Unimproved | 1.35 (1.11-1.63)* | 1.32 (1.08-1.62)* | 1.35 (1.17-1.55)** | 1.07 (0.85-1.34) | 1.33 (1.01-1.74)* | 1.18 (0.99-1.40) | 1.63 (1.32-2.01)** | 1.36 (1.08-1.72)* | 1.51 (1.29-1.76)** |
| **Source of drinking water** |  |  |  |  |  |  |  |  |  |
| Improved | Ref. | Ref. | Ref. | Ref. | Ref. | Ref. | Ref. | Ref. | Ref. |
| Unimproved | 1.08 (0.94-1.23) | 0.88 (0.77-1.02) | 0.98 (0.88-1.08) | 0.98 (0.84-1.14) | 0.80 (0.67-0.95)* | 0.89 (0.79-0.99)* | 0.97 (0.84-1.12) | 0.90 (0.78-1.05) | 0.93 (0.84-1.03) |
| **Time to get a water source** |  |  |  |  |  |  |  |  |  |
| On-premise | Ref. | Ref. | Ref. | Ref. | Ref. | Ref. | Ref. | Ref. | Ref. |
| ≤ 30 min | 0.98 (0.77-1.26) | 1.30 (0.99-1.69) | 1.13 (0.94-1.35) | 1.03 (0.77-1.38) | 1.02 (0.72-1.42) | 1.02 (0.82-1.28) | 0.83 (0.63-1.08) | 1.31 (0.96-1.77) | 1.02 (0.83-1.25) |
| 31-60 min | 1.05 (0.80-1.37) | 1.19 (0.89-1.60) | 1.12 (0.92-1.37) | 1.08 (0.79-1.48) | 1.09 (0.76-1.57) | 1.09 (0.86-1.38) | 0.85 (0.63-1.14) | 1.13 (0.81-1.57) | 0.97 (0.78-1.21) |
| >60 min | 1.01 (0.77-1.33) | 1.27 (0.95-1.72) | 1.14 (0.93-1.39) | 1.29 (0.94-1.76) | 1.30 (0.90-1.87) | 1.29 (1.02-1.64)* | 0.87 (0.65-1.18) | 1.45 (1.04-2.02)* | 1.09 (0.88-1.37) |
| ***Community-level characteristics*** |  |  |  |  |  |  |  |  |  |
| **Residence** |  |  |  |  |  |  |  |  |  |
| Urban | 0.67 (0.53-0.84)* | 0.77 (0.60-0.98)* | 0.72 (0.61-0.85)** | 1.57 (1.21-2.04)* | 1.41 (1.04-1.92)* | 1.48 (1.21-1.81)** | 0.72 (0.56-0.93)* | 1.03 (0.78-1.34) | 0.84 (0.69-1.02) |
| Rural | Ref. | Ref. | Ref. | Ref. | Ref. | Ref. | Ref. | Ref. | Ref. |
| **Region** |  |  |  |  |  |  |  |  |  |
| Agrarian | Ref. | Ref. | Ref. | Ref. | Ref. | Ref. | Ref. | Ref. | Ref. |
| Pastoralist | 0.87 (0.76-1.01) | 1.03 (0.89-1.20) | 0.94 (0.85-1.05) | 1.04 (0.90-1.22) | 1.01 (0.85-1.20) | 1.02 (0.91-1.15) | 1.14 (0.98-1.32) | 0.95 (0.81-1.10) | 1.11 (0.99-1.24) |
| City administration | 0.70 (0.57-0.86)* | 0.84 (0.68-1.03) | 0.77 (0.66-0.89)* | 0.98 (0.78-1.23) | 0.97 (0.75-1.25) | 0.97 (0.82-1.16) | 0.98 (0.77-1.25) | 0.83 (0.66-1.05) | 0.93 (0.77-1.11) |
| **EDHS** |  |  |  |  |  |  |  |  |  |
| 2000 | 1.72 (1.64-1.89)* | 1.51 (1.14-1.77)* | 1.49 (1.32-1.91)* | 1.12 (0.74-1.29) | 1.72 (0.51-1.98) | 1.31 (0.09-1.40) | 1.57 (1.43-1.72)* | 1.33 (1.24-1.67)* | 1.48 (1.41-1.86)* |
| 2005 | 1.68 (1.41-2.03)* | 1.44 (1.19-1.73)** | 1.56 (1.36-1.78)** | 1.09 (0.89-1.34) | 0.94 (0.75-1.17) | 1.08 (0.87-1.18) | 1.41 (1.17-1.70)** | 1.22 (1.05-1.49)* | 1.31 (1.14-1.50)** |
| 2011 | 1.27 (1.10-1.45)* | 1.15 (0.99-1.32) | 1.21 (1.09-1.34)** | 1.03 (0.88-1.21) | 0.91 (0.76-1.08) | 0.97 (0.86-1.09) | 1.39 (1.20-1.60)** | 1.18 (1.02-1.38)* | 1.28 (1.15-1.43)** |
| 2016 | Ref. | Ref. | Ref. | Ref. | Ref. | Ref. | Ref. | Ref. | Ref. |
| **Random effect** |  |  |  |  |  |  |  |  |  |
| Variance (SE) | 0.0971 (0.0031)** | 0.0872 (0.0038)* | 0.1018 (0.0011)*** | 0.0877 (0.0045)*** | 0.1187 (0.0052)* | 0.1022 (0.0016)*** | 0.1073 (0.0034)** | 0.0663(0.0052)* | 0.0928 (0.0012)*** |
| ICC | 2.86 | 2.58 | 3.00 | 2.59 | 3.48 | 3.01 | 3.15 | 1.97 | 2.74 |
| **Model fitness** |  |  |  |  |  |  |  |  |  |
| AIC | 7530.55 | 7105.74 | 14585.27 | 6988.41 | 5856.96 | 12795.49 | 7248.70 | 6524.17 | 13731.53 |
| BIC | 7795.59 | 7369.34 | 14884.1 | 7251.12 | 6118.52 | 13091.05 | 7507.34 | 6781.37 | 14023.28 |
